# Supplementary material for: Atomically precise gold nanoclusters at the molecular-to-metallic transition with intrinsic chirality from surface layers
Source: Nat Commun. 2023 Apr 26;14:2397. doi: 10.1038/s41467-023-38179-0 (PMC10133330; doi:10.1038/s41467-023-38179-0)

Supplementary Information

Atomically precise gold nanoclusters at the molecular-to-metallic transition with intrinsic chirality from surface layers

Li-Juan Liu[1], Fahri Alkan[2], Shengli Zhuang[1,3], Dongyi Liu[1], Tehseen Nawaz[1], Jun Guo[1], Xiaozhou Luo[4], and Jian He[1,3]*

[1]Department of Chemistry, The University of Hong Kong, Hong Kong, China.

[2]Department of Nanotechnology Engineering, Abdullah Gül University, Kayseri, Turkey.

[3]State Key Laboratory of Synthetic Chemistry, The University of Hong Kong, Hong Kong, China.

[4]Center for Synthetic Biochemistry, Shenzhen Institute of Synthetic Biology, Shenzhen Institutes of Advanced Technology, Chinese Academy of Sciences, Shenzhen, China.

Email: jianhe@hku.hk

Table of Contents

1. Supplementary figures S–2

2. X-ray crystallography S–19

3. Supplementary references S–26

4. CheckCIF report and explanations S–26

1. Supplementary figures

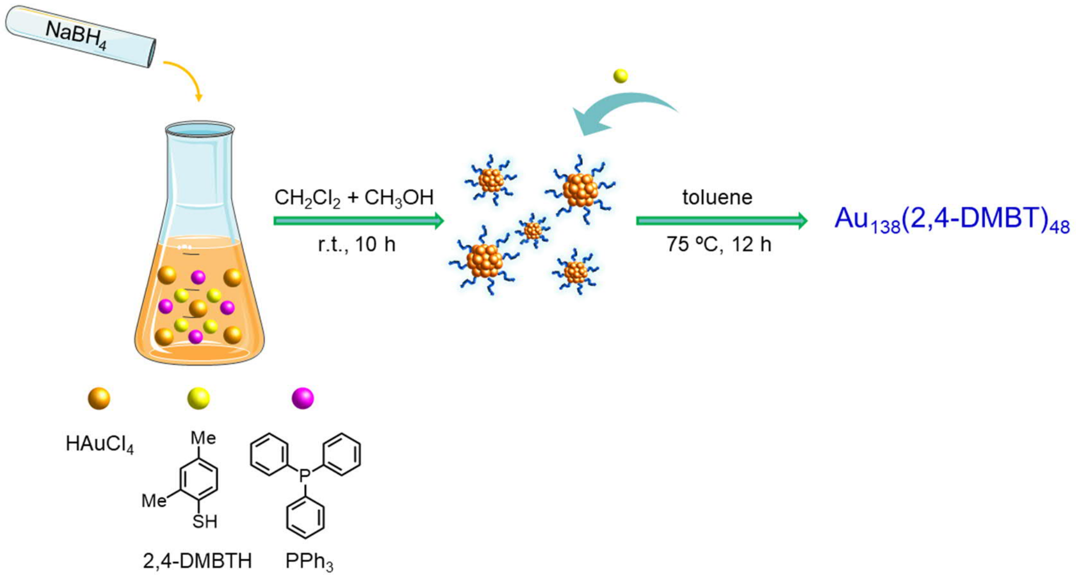

Supplementary Figure 1. The synthesis scheme for $Au_{138}(SR)_{48}$. R = 2,4-$Me_2C_6H_3$.

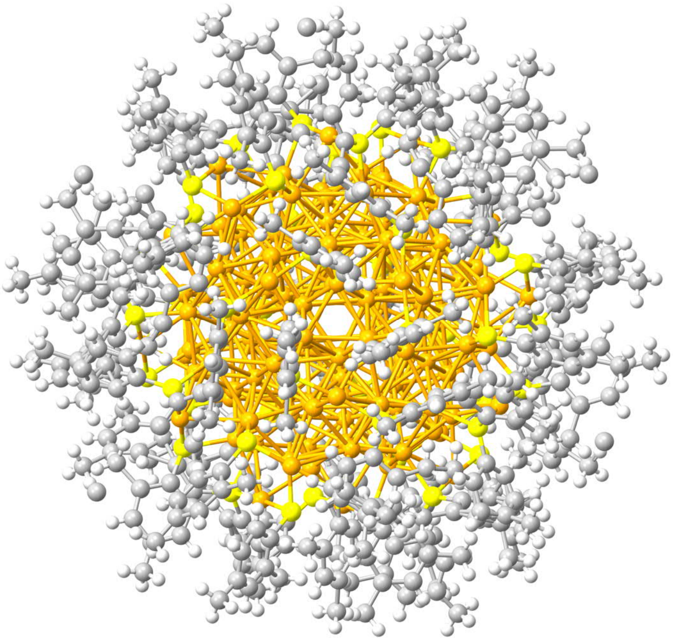

Supplementary Figure 2. Molecular structure of $Au_{138}(SR)_{48}$ viewed along the [111] direction.

Color labels: Au, orange; S, yellow; C, gray; H, white.

3).

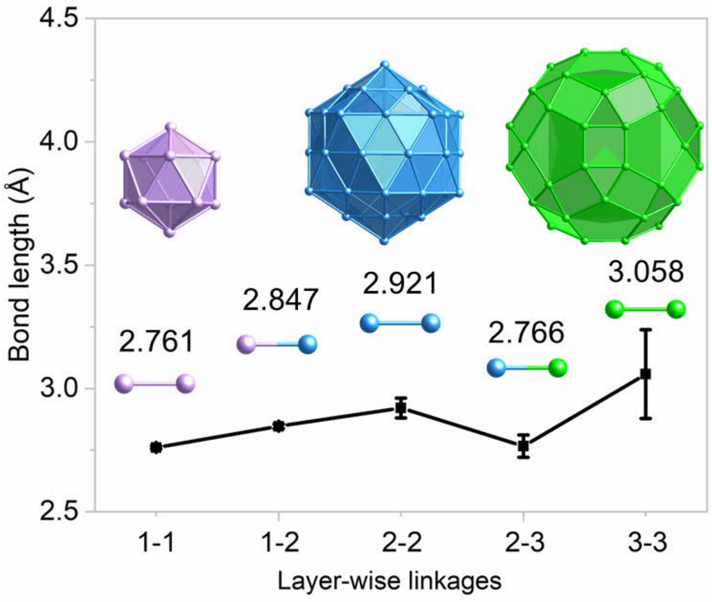

Supplementary Figure 3. Average Au−Au bond length: comparison from shell 1 to shell 3 in $Au_{138}(SR)_{48}$. X-axis: 1-1 − within shell 1; 1-2 − between shell 1 and shell 2; 2-2 − within shell 2; 2-3 − between shell 2 and shell 3; 3-3 − within shell 3. Color labels of Au: pink (from shell 1); blue (from shell 2); green (from shell 3).

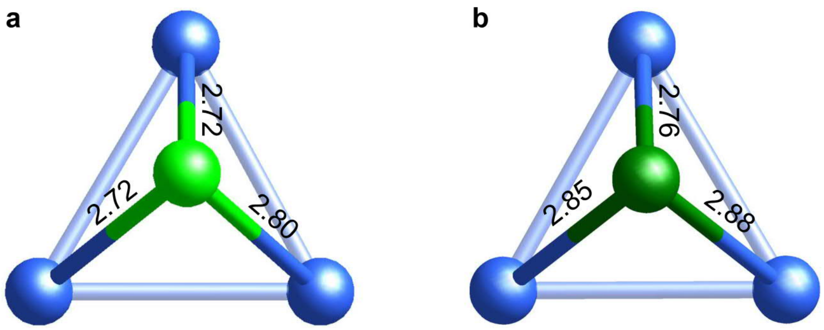

Supplementary Figure 4. Comparison of the Au−Au bond lengths between shell 2 and shell 3 in $Au_{138}(SR)_{48}$ and $Au_{144}(SCH_2Ph)_{60}$[1]. **a** The distance between the exposed gold atom and the gold atoms from the closest triangle of the second shell in $Au_{138}(SR)_{48}$. **b** The distance between the gold atom in the third shell and the gold atoms from the closest triangle of the second shell in $Au_{144}(SCH_2Ph)_{60}$. Color labels of Au: blue (from shell 2); bright green and green (from shell 3).

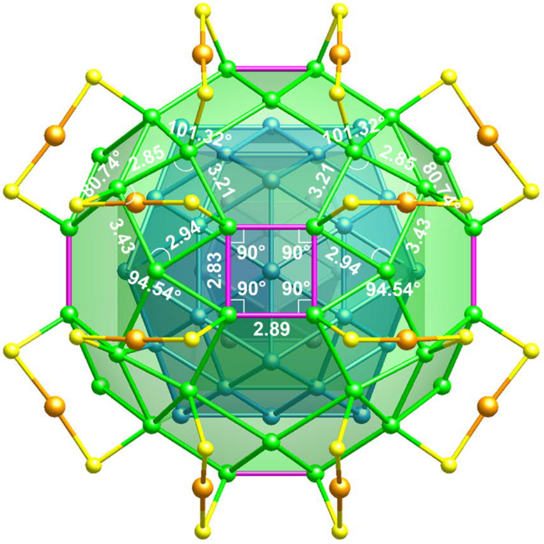

Supplementary Figure 5. Au−Au bond lengths and angles in the Au_{60} shell. Color labels: Au, pink, blue, green, and orange; S, yellow. Aryl groups on thiolates are omitted for clarity.

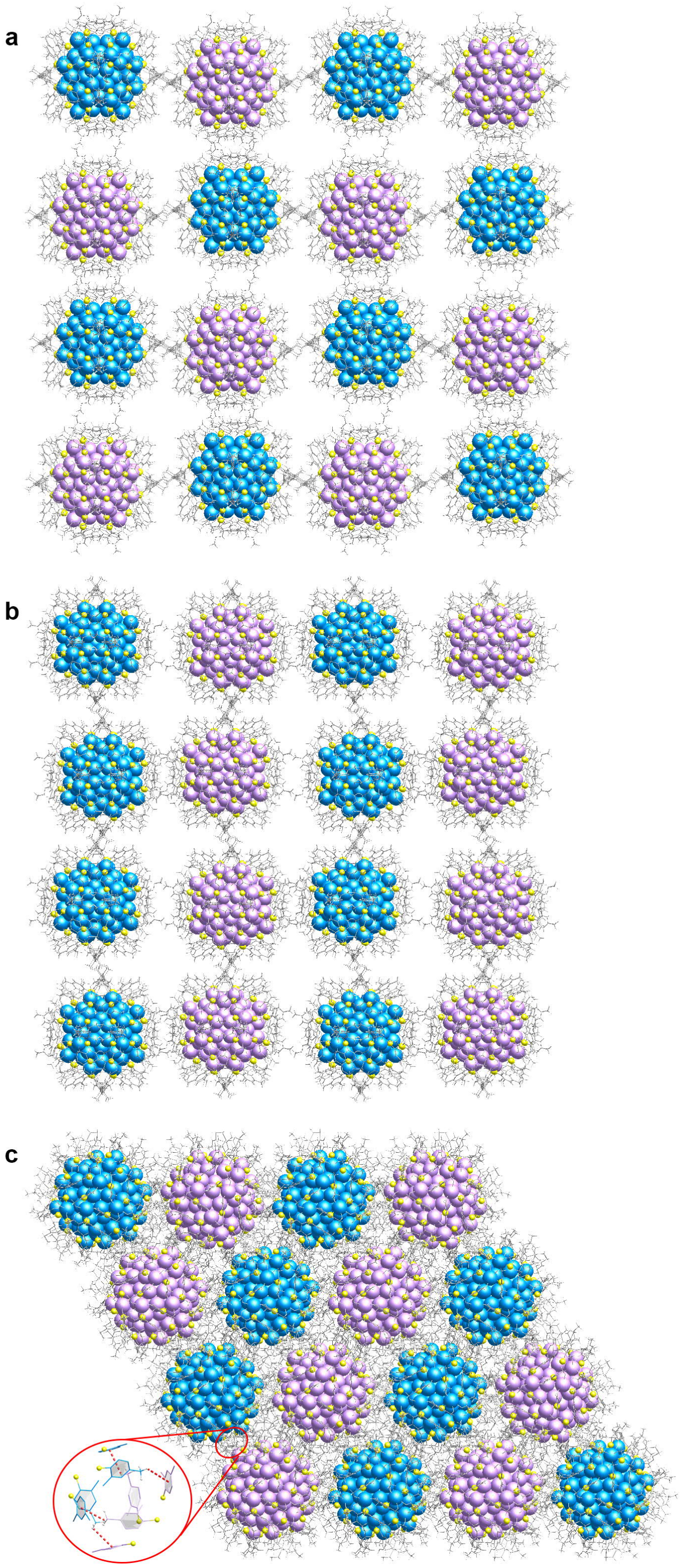

Supplementary Figure 6. Packing structure of $Au_{138}(SR)_{48}$ in single crystals. Views along the [100] (**a**), [010] (**b**), and [111] (**c**) directions. Inset: inter-cluster C−H···π interactions between the protective shells. Color labels: Au, pink, blue (indicating two different layers viewed along the [010] direction); S, yellow; C, grey; H white.

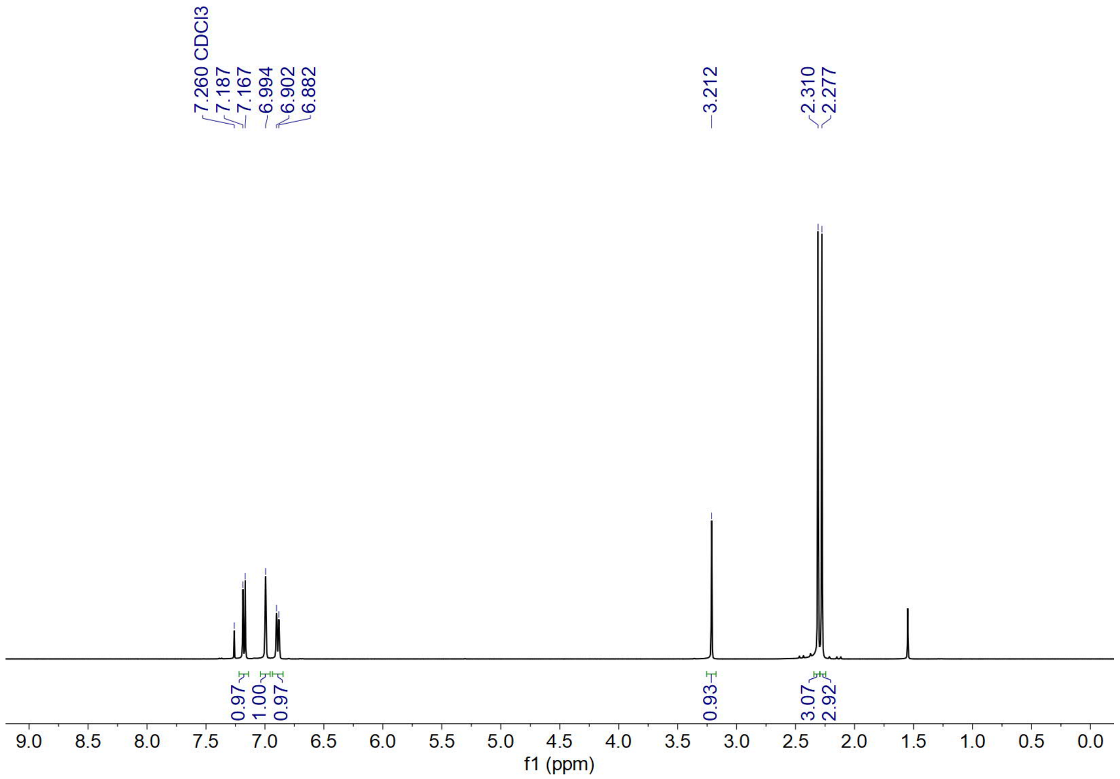

Supplementary Figure 7. ^{1}H NMR spectrum of free 2,4-DMBTH.

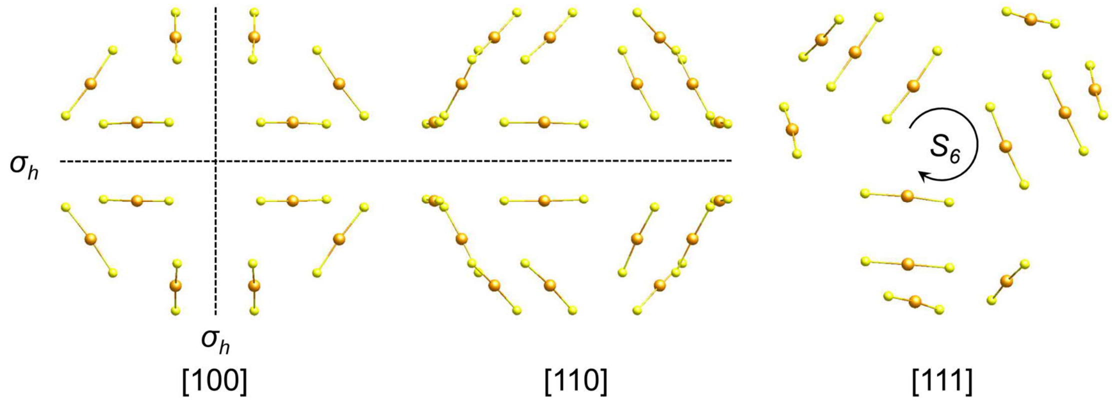

Supplementary Figure 8. Views of the interfacial layer of $Au_{138}(SR)_{48}$. Color labels: Au, orange; S, yellow.

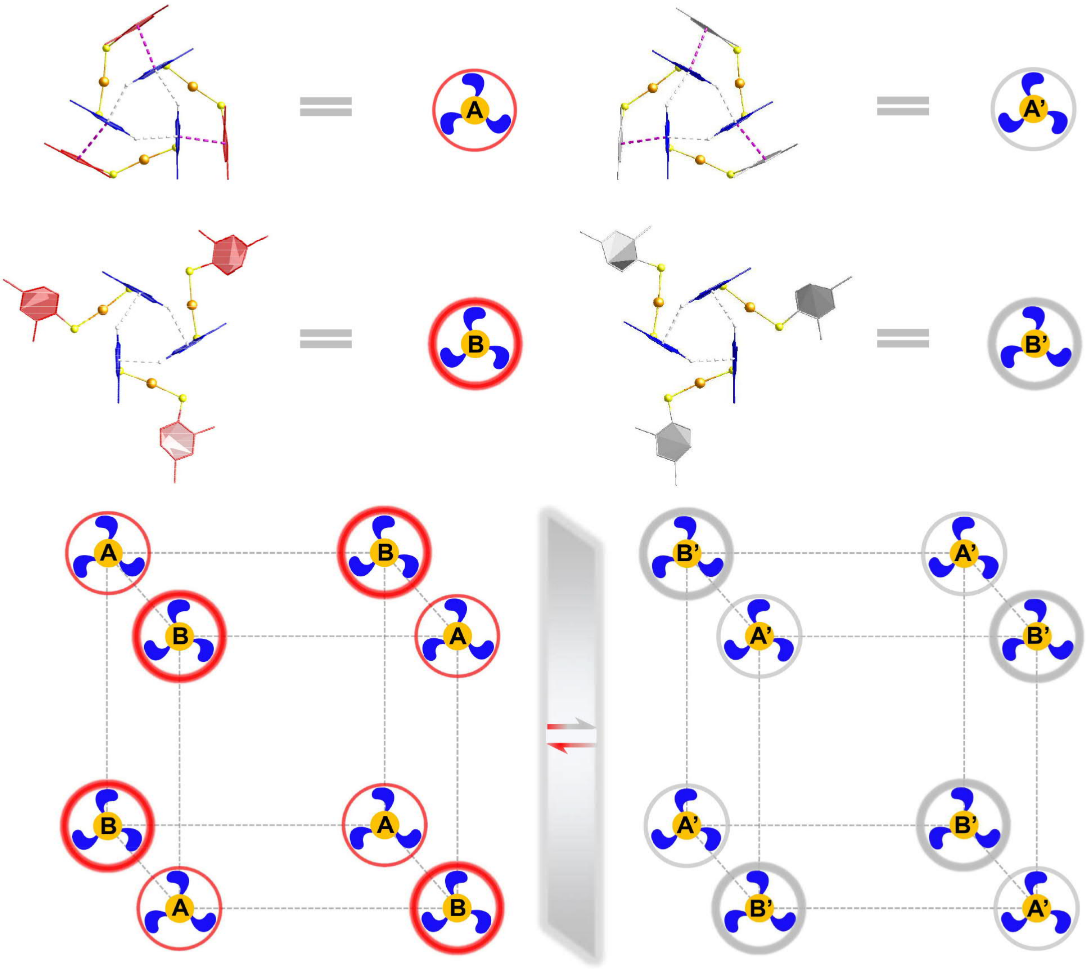

Supplementary Figure 9. Schematic illustration for enantiomeric distributions of aryl groups on the [−SR−Au−SR−] staples. The thin and thick circles with three fan blades inside depict the protective units with and without π-π stacking, respectively. The directions of the blue fan blades reflect the orientations of the aryl groups containing C−H···π interactions; the red and gray circles represent the aryl groups on the periphery of the protective units from two enantiomers of $Au_{138}(SR)_{48}$. Color labels: Au, orange; S, yellow; C, red and blue; H, white. Staples from the back are omitted for clarity.

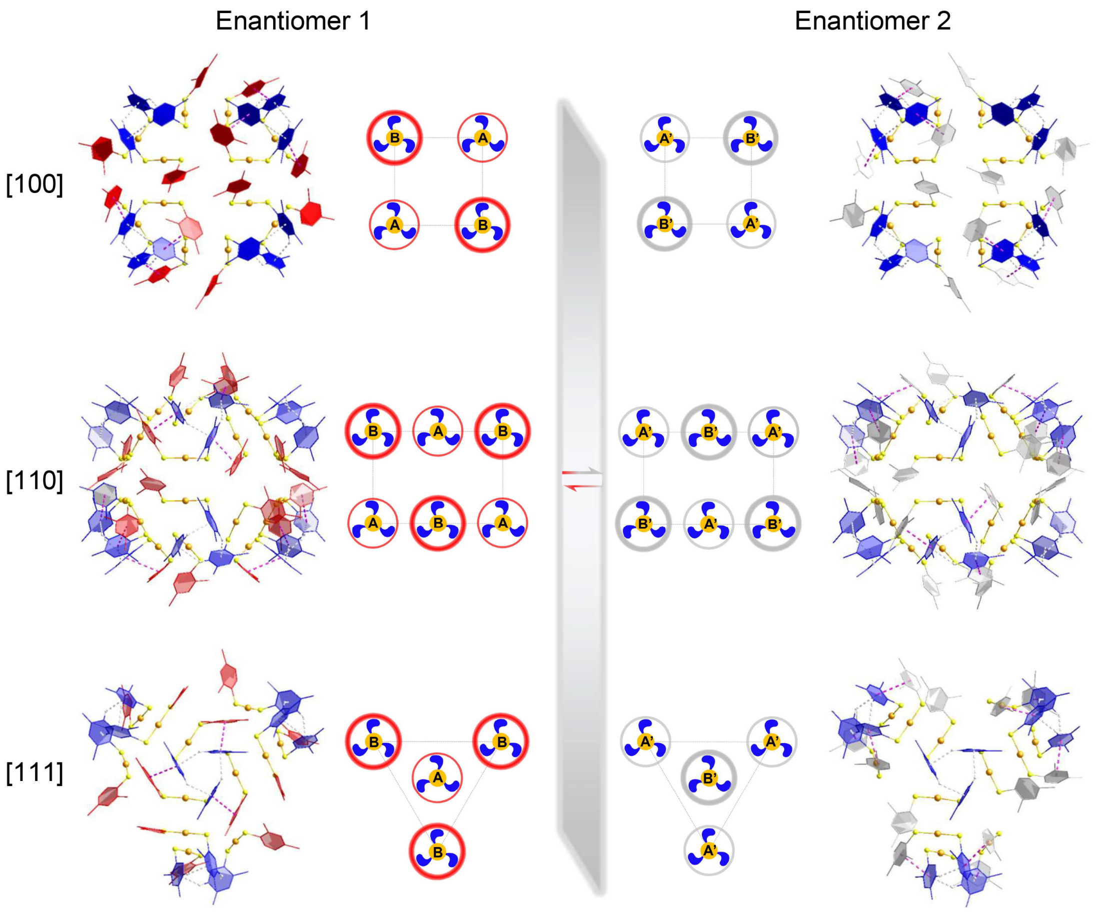

Supplementary Figure 10. Enantiomeric distributions of aryl groups on the [−SR−Au−SR−] staples from different views. The thin and thick circles with three fan blades inside depict the protective units with and without π−π stacking, respectively. The directions of the blue fan blades reflect the orientations of the aryl groups containing C−H···π interactions; the red and gray circles represent the aryl groups on the periphery of the protective units from two enantiomers of $Au_{138}(SR)_{48}$. Color labels: Au, orange; S, yellow; C, red and blue; H, white. Staples from the back are omitted for clarity.

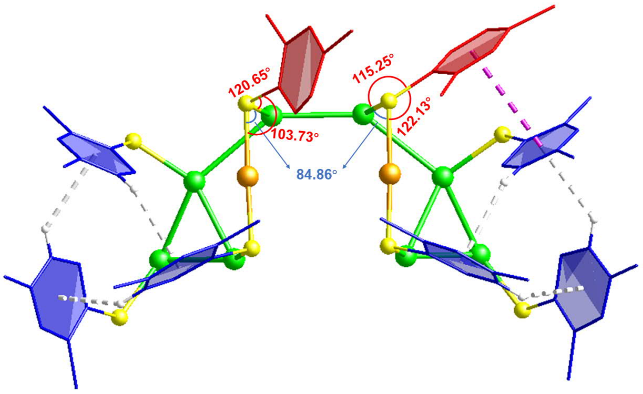

Supplementary Figure 11. Detail view profile of the [Au−S−Au−S−Au]$_2$ motif showing different hybridization characteristics of the sulfur atoms. Color labels: Au, green and orange; S, yellow; C, red and blue; H, white.

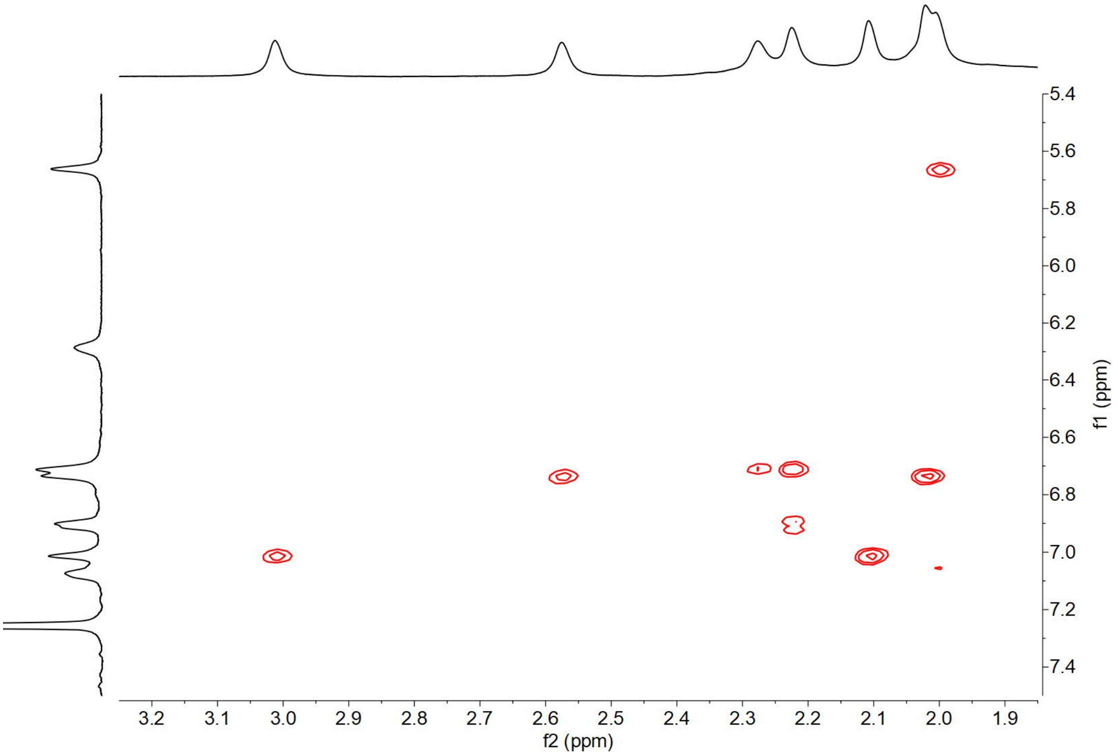

Supplementary Figure 12. ^1H–^1H COSY spectrum of $Au_{138}(SR)_{48}$ showing long-range couplings with aliphatic protons.

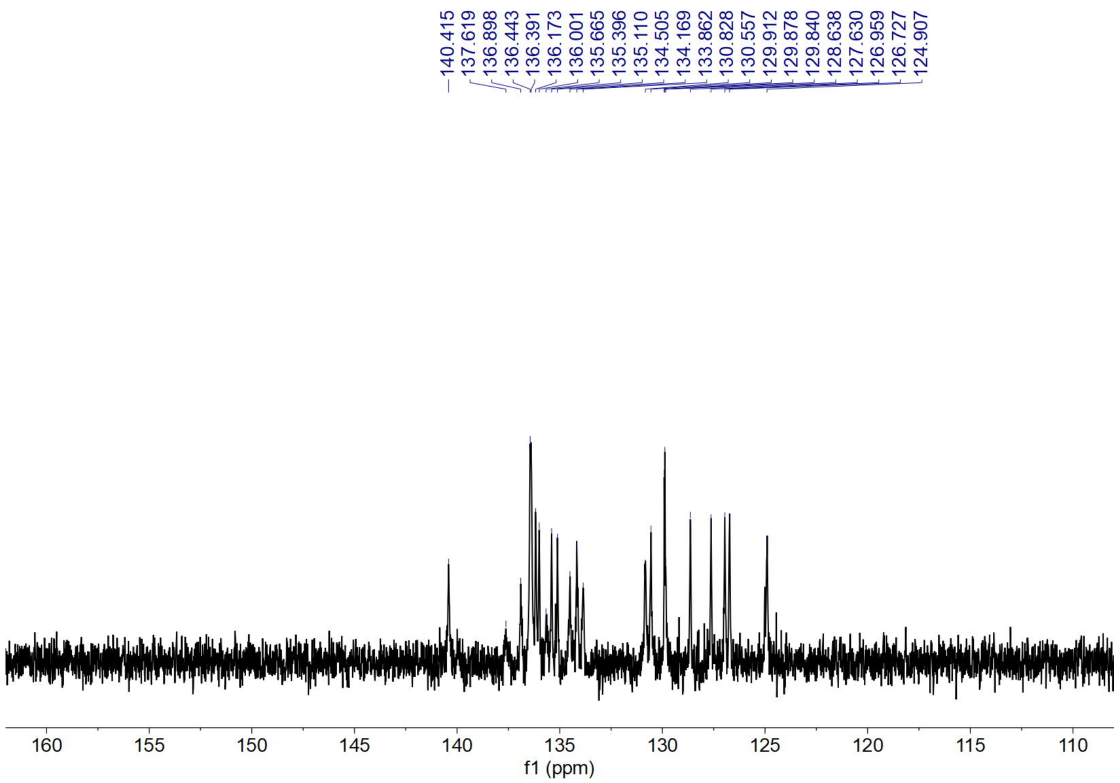

Supplementary Figure 13. ^{13}C NMR spectrum of $Au_{138}(SR)_{48}$.

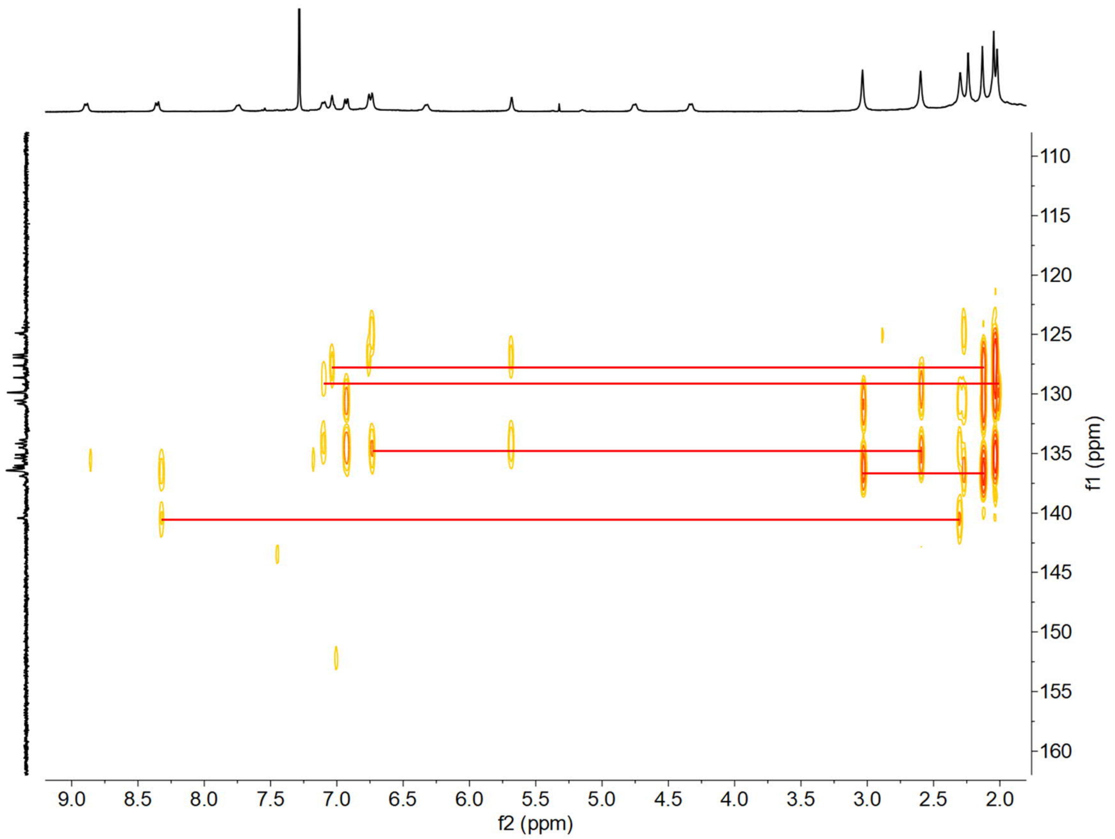

Supplementary Figure 14. HMBC spectrum of $Au_{138}(SR)_{48}$.

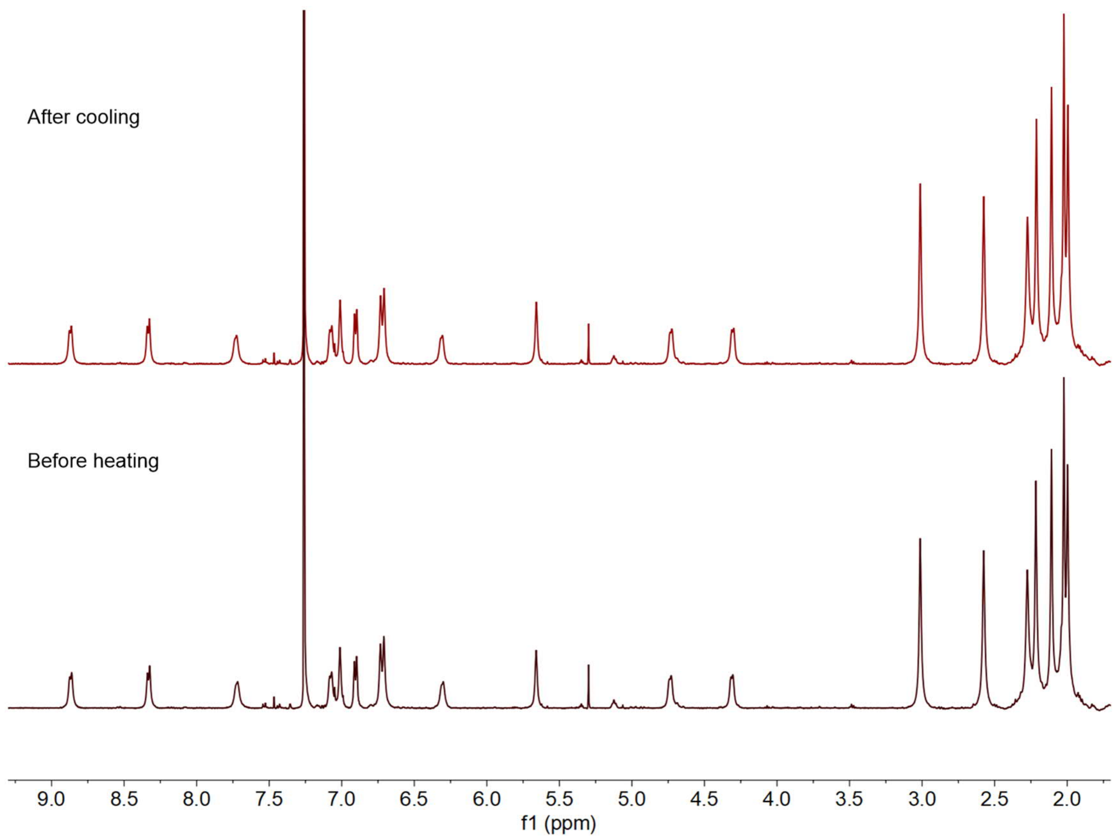

Supplementary Figure 15. ^{1}H NMR spectrum of $Au_{138}(SR)_{48}$ before heating and after cooling to 298 K from 328 K.

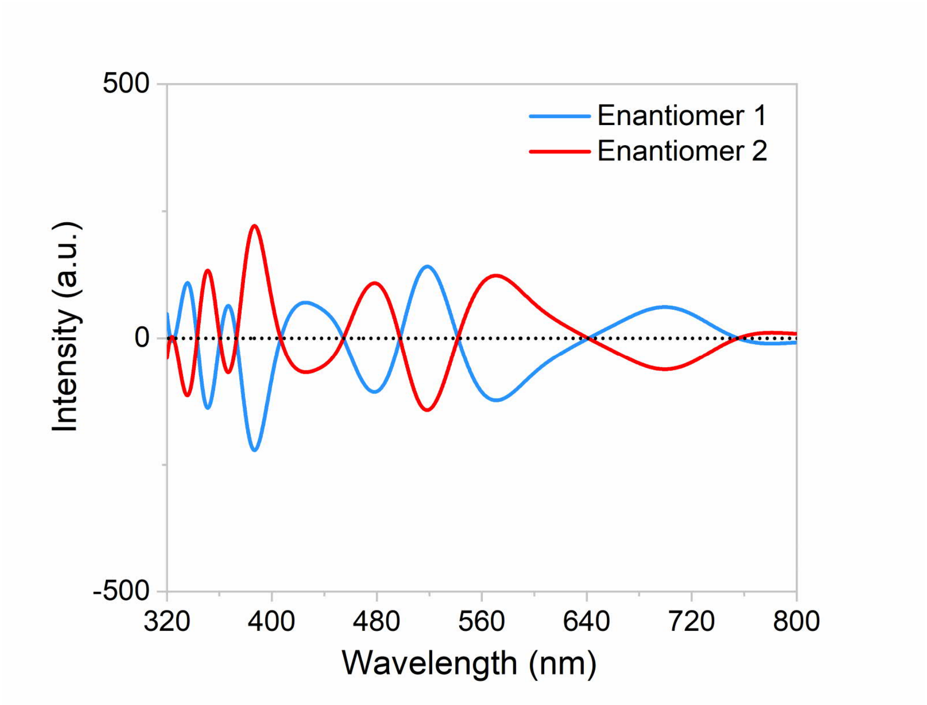

Supplementary Figure 16. Calculated circular dichroism (CD) spectra for the enantiomers of $Au_{138}(SH)_{48}$ with a *T* symmetry. Source data are provided as a Source Data file.

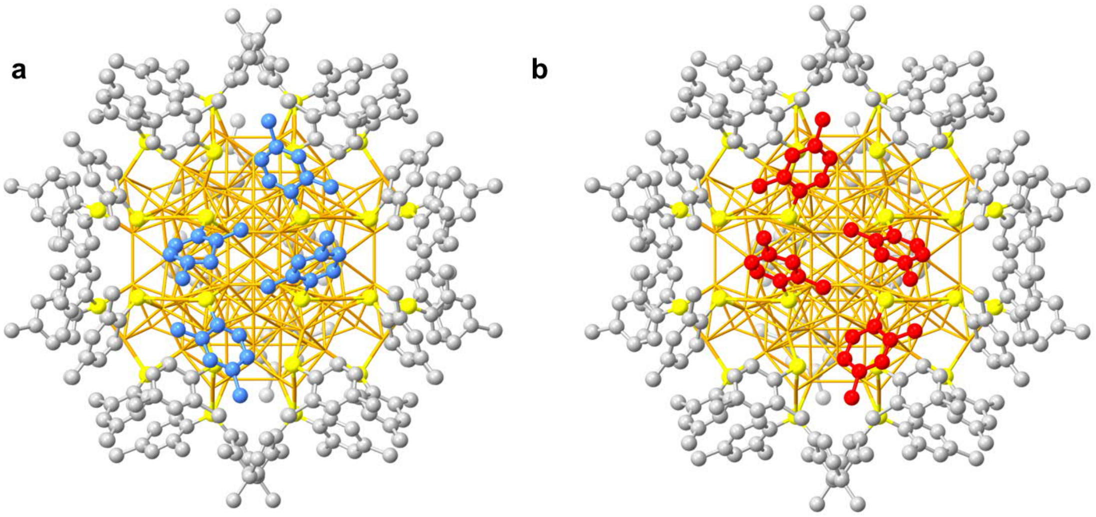

Supplementary Figure 17. Optimized structure for the enantiomers of $Au_{138}(SR)_{48}$ with a *T* symmetry. **a** Calculated Enantiomer 1 with a CD spectrum presented in blue in Fig. 4b. **b** Calculated Enantiomer 2 with a CD spectrum presented in red in Fig. 4b. Color labels: Au, orange; S, yellow; C, gray, blue, and red.

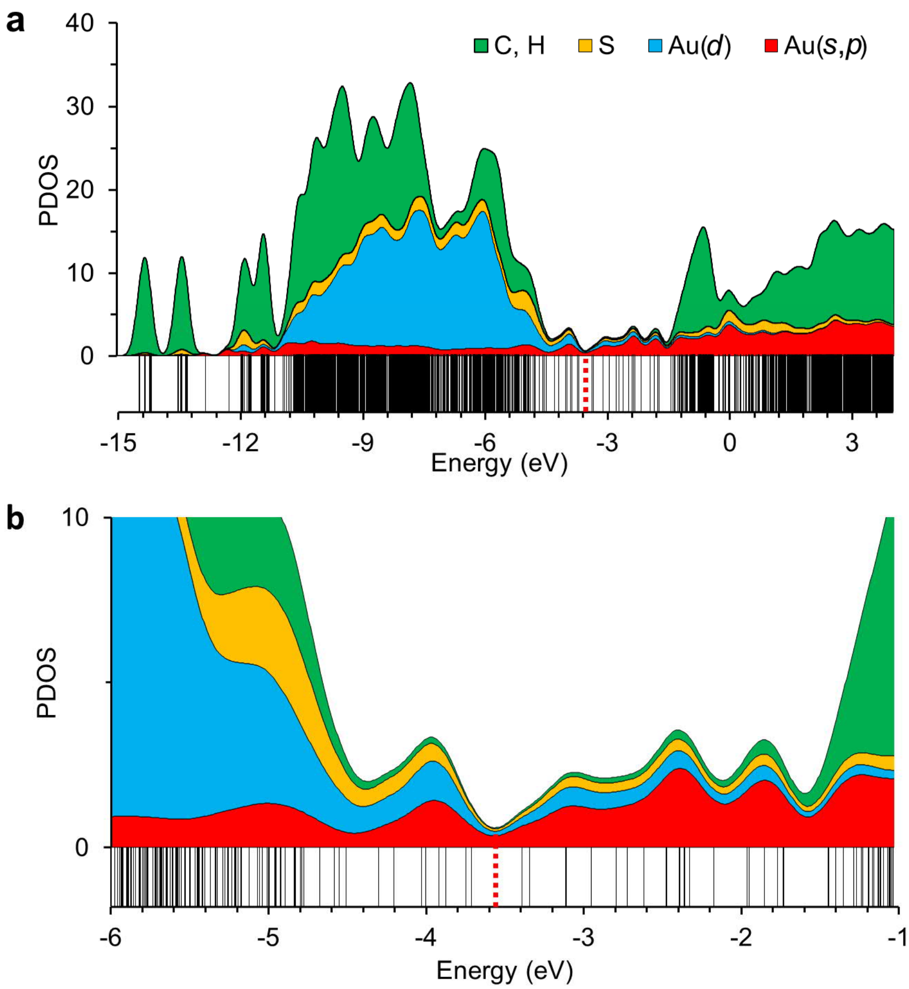

Supplementary Figure 18. Calculated partial and total density of states curves for $Au_{138}(SR)_{48}$ obtained by PBE/SVP level of theory. For Au atoms, contributions from *sp* and *d* bands are shown separately in red and blue. **a** Partial density of states (PDOS) curves for the energy range between -15 and 4 eV. **b** The same curves for a smaller energy range for clarity with a focus on the contributions to frontier levels. Source data are provided as a Source Data file.

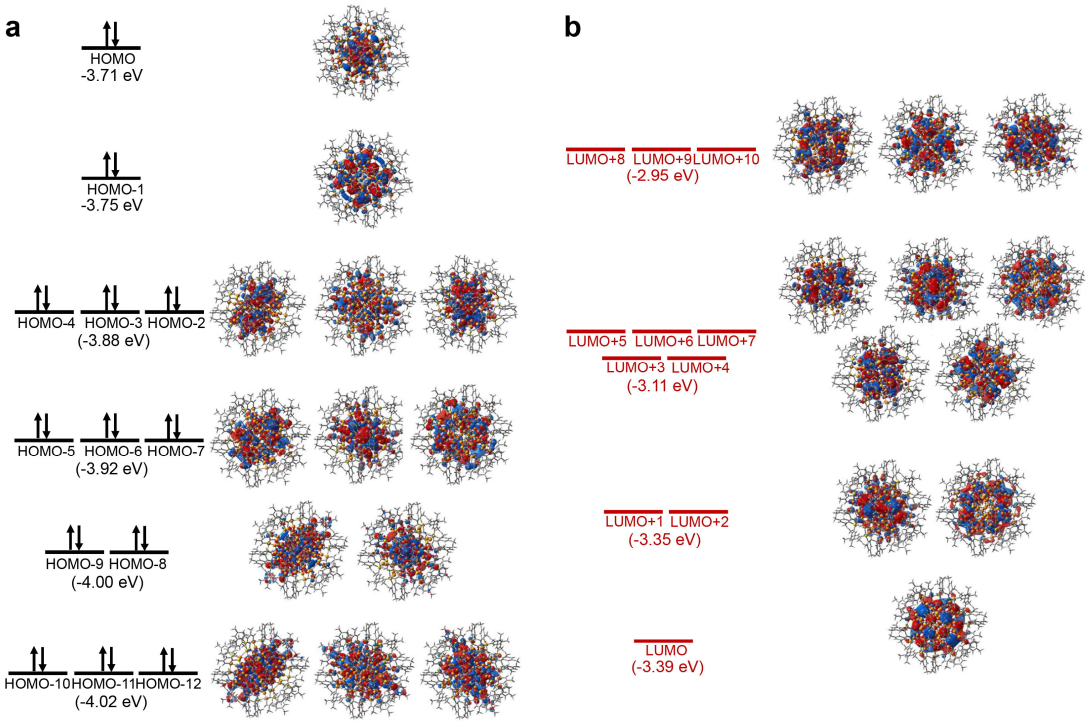

Supplementary Figure 19. Energies and pictorial representations of occupied (**a**) and unoccupied (**b**) frontier molecular orbitals of $Au_{138}(SR)_{48}$ obtained by PBE/SVP level of theory. Source data are provided as a Source Data file.

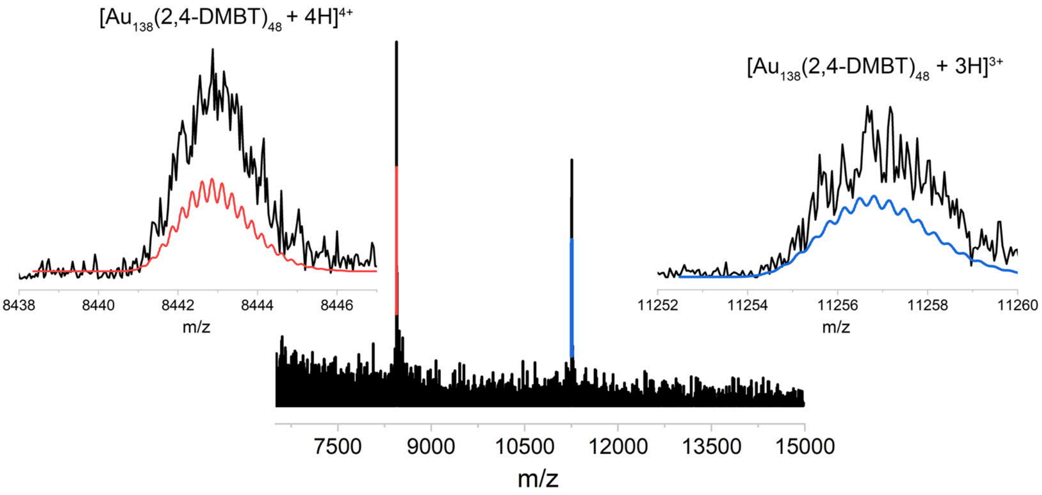

Supplementary Figure 20. Electrospray ionization time-of-flight mass spectra of $Au_{138}(SR)_{48}$. Insets: the measured (black line) and simulated (red or blue line) isotopic patterns. Source data are provided as a Source Data file.

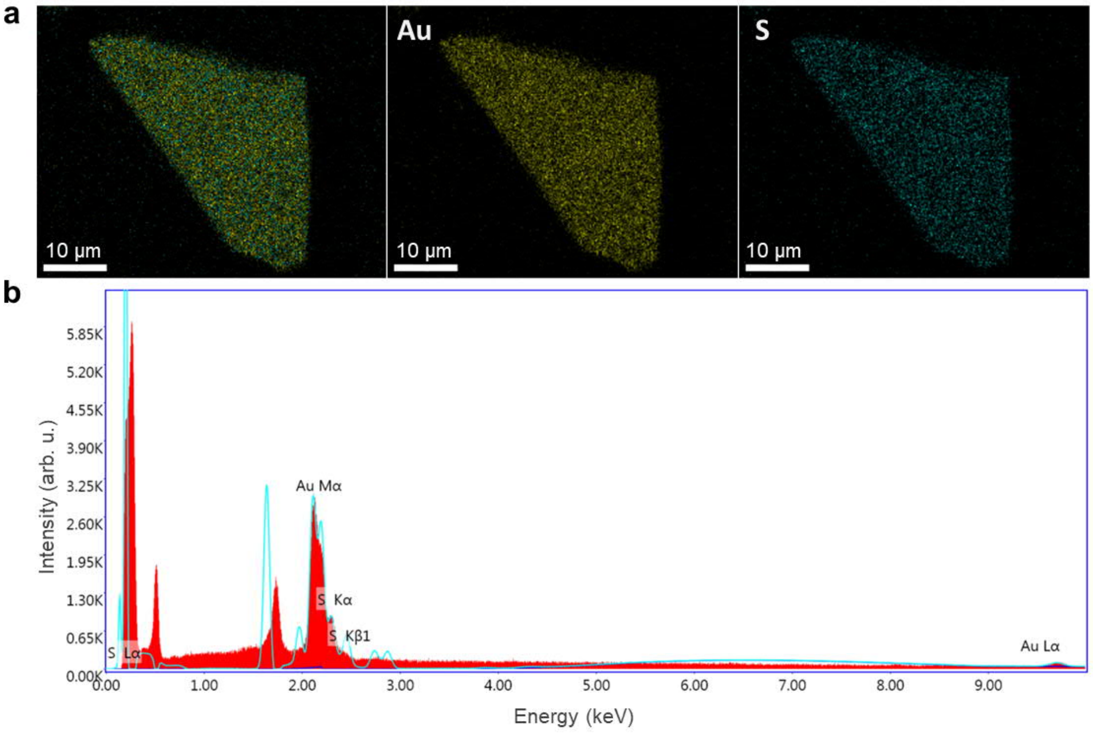

Supplementary Figure 21. Energy dispersive spectroscopy (**a**) and mapping (**b**) of $Au_{138}(SR)_{48}$. Color labels: Au, yellow; S, blue.

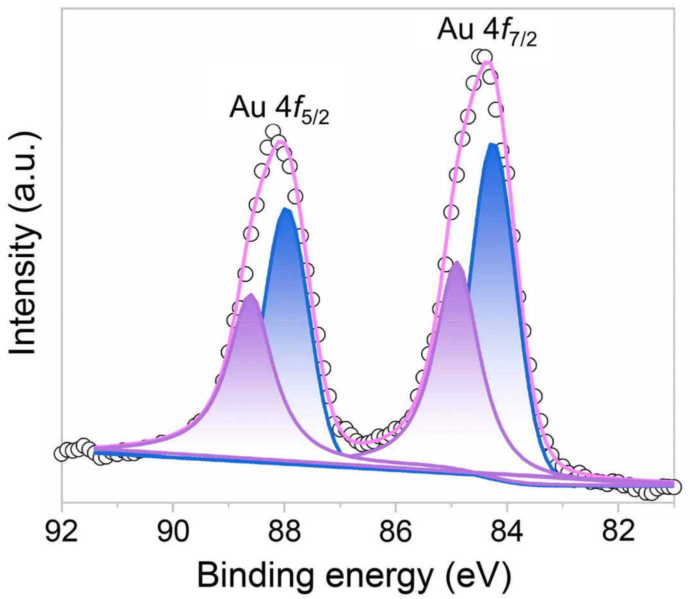

Supplementary Figure 22. High-resolution XPS spectrum of Au 4*f* in $Au_{138}(SR)_{48}$ indicating the presence of Au(0) and Au(I). The blue and purple peaks were assigned to Au(0) and Au(I) species, respectively.

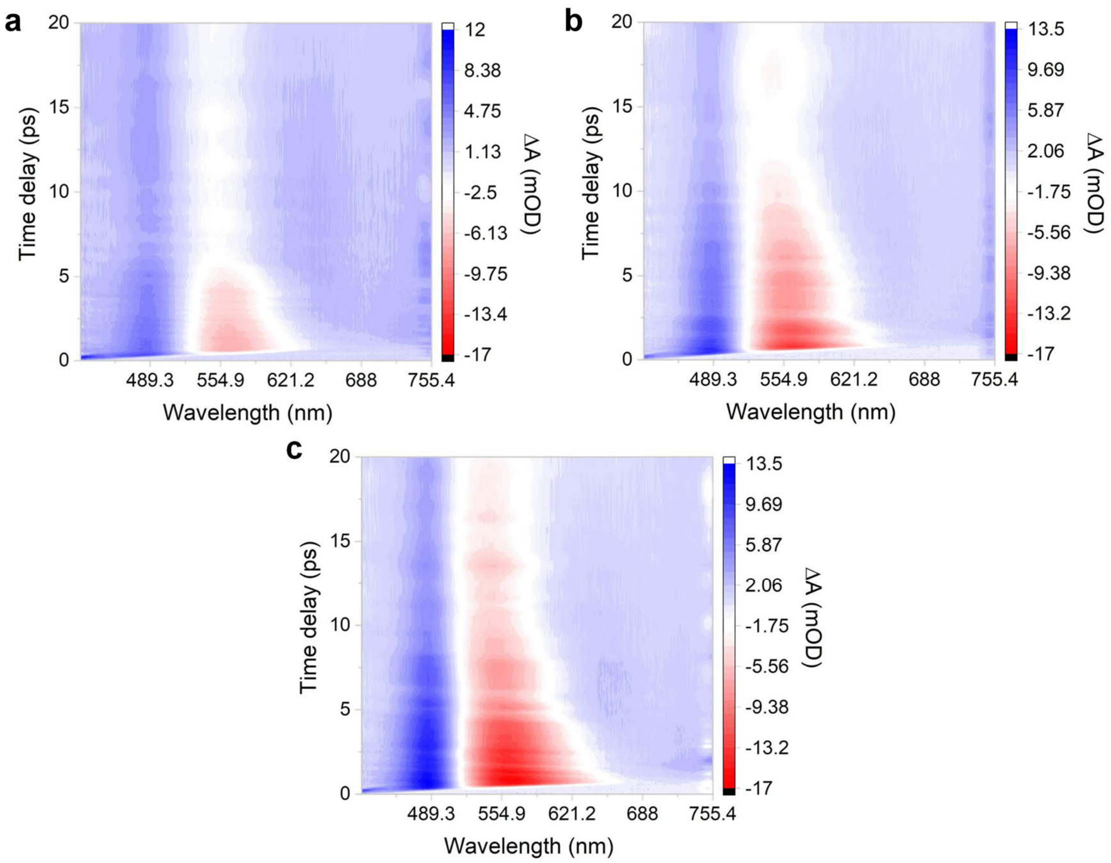

Supplementary Figure 23. Transient absorption data maps pumped at 380 nm with pump power varying from 70 μW pulse^{-1} (**a**), 80 μW pulse^{-1} (**b**), and 90 μW pulse^{-1} (**c**). Source data are provided as a Source Data file.

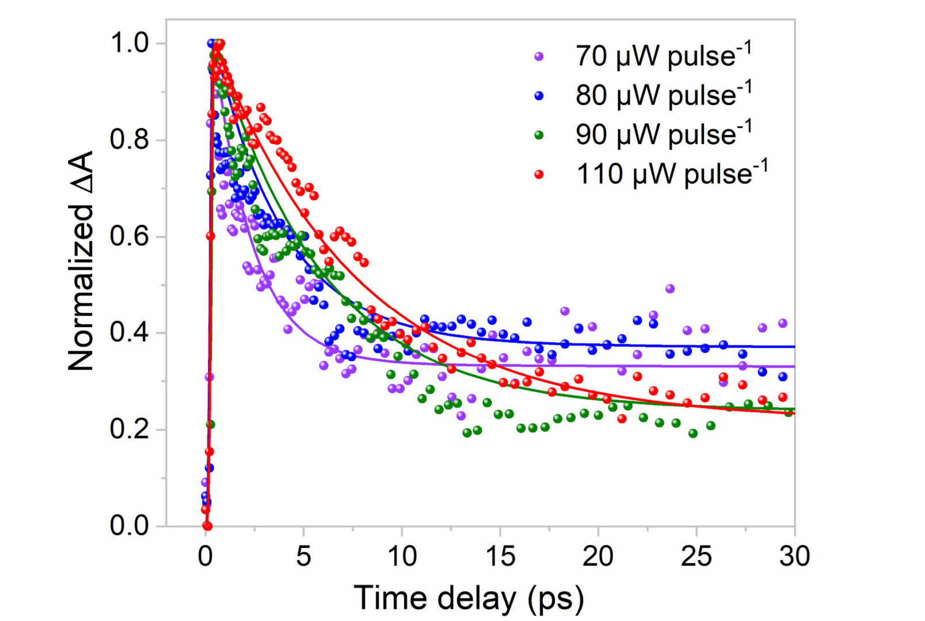

Supplementary Figure 24. Normalized decay kinetics around 485 nm as a function of laser fluence. Source data are provided as a Source Data file.

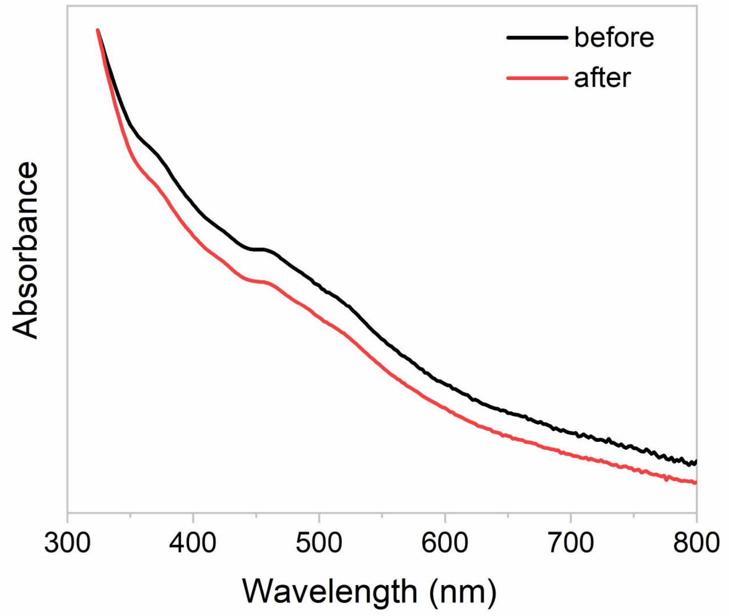

Supplementary Figure 25. UV-vis absorption spectra of $Au_{138}(SR)_{48}$ in toluene before and after femtosecond transient absorption measurements. Source data are provided as a Source Data file.

2. X-ray crystallography

Supplementary Table 1. Crystal data and structure refinement for $Au_{138}(SR)_{48}$

Identification code	$Au_{138}(SR)_{48}$
CCDC number	2195004
Empirical formula	$C_{384}H_{432}Au_{138}S_{48}$
Formula weight	33767.4
Temperature/K	173
Crystal system	cubic
Space group	*F*m-3c
a/Å	52.7982(8)
b/Å	52.7982(8)
c/Å	52.7982(8)
α/°	90
β/°	90
γ/°	90
Volume/Å^3	147183(7)
Z	8
ρ_{calc} g/cm^3	3.008
μ/mm^{-1}	51.665
F(000)	111793.0
Crystal size/mm^3	0.005 × 0.005 × 0.005
Radiation	CuKα (λ = 1.54178)
2θ range for data collection/°	4.734 to 133.772
Index ranges	$-62 \leq h \leq 62$, $-62 \leq k \leq 62$, $-62 \leq l \leq 51$
Reflections collected	314234
Independent reflections	5676 [R_{int} = 0.1665, R_{sigma} = 0.0323]
Data/restraints/parameters	5676/498/287
Goodness-of-fit on F^2	1.223
Final R indexes [I >= 2σ (I)]	R_1 = 0.0567, wR_2 = 0.1580
Final R indexes [all data]	R_1 = 0.0717, wR_2 = 0.1685
Largest diff. peak/hole/eÅ^{-3}	1.99/−3.77

$R_1 = \sum||F_o|-|F_c||/\sum|F_o|$. $wR_2 = [\sum w(F_o^2-F_c^2)^2/\sum w(F_o^2)^2]^{1/2}$

Supplementary Table 2. Bond lengths in $Au_{138}(SR)_{48}$

Bond	Length/Å	Bond	Length/Å
Au1–Au1^{1}	2.745(2)	Au1–Au1^{2}	2.7651(13)
Au1–Au2^{5}	2.8592(18)	Au1–Au3	2.8407(11)
Au1–Au3^{6}	2.8480(13)	Au1–Au4^{4}	2.9334(17)
Au2–Au1^{3}	2.8593(18)	Au2–Au3^{3}	2.9595(10)
Au2–Au3^{4}	2.9595(10)	Au2–Au4^{2}	2.9609(12)
Au2–Au5^{9}	2.7569(14)	Au3–Au1^{2}	2.8478(13)
Au3–Au2^{5}	2.9595(10)	Au3–Au3^{11}	2.9802(17)
Au3–Au3^{2}	2.8493(15)	Au3–Au4^{4}	2.9363(11)
Au3–Au5^{2}	2.9845(12)	Au3–Au4	2.8903(13)
Au3–Au7	2.7455(13)	Au3–Au6	2.7167(13)
Au4–Au1^{2}	2.9334(17)	Au3–Au7^{2}	2.9308(13)
Au4–Au3^{2}	2.9364(11)	Au4–Au2^{3}	2.9609(12)
Au4–Au3^{11}	2.8903(13)	Au4–Au5^{6}	2.8255(14)
Au4–Au6	2.7995(18)	Au4−Au7^{2}	2.8930(11)
Au5–Au3^{6}	2.9845(12)	Au5–Au4^{2}	2.8253(14)
Au5–Au5^{10}	2.8315(19)	Au5–Au5^{7}	2.885(2)
Au5–Au6^{4}	2.9370(16)	Au5–Au7^{6}	3.2114(14)
Au5–Au8	3.1614(15)	Au6–Au3^{11}	2.7166(13)
Au6–Au5^{2}	2.9370(16)	Au6–Au5^{12}	2.9370(16)
Au6–Au8^{12}	2.7806(13)	Au6–Au8^{2}	2.7806(13)
Au7–Au3^{6}	2.9308(13)	Au7–Au4^{4}	2.8931(11)
Au7–Au5^{2}	3.2114(13)	Au7–Au7^{2}	2.8477(17)
Au7–Au7^{6}	2.8478(17)	Au7–Au8	3.2625(15)
Au7–Au8^{2}	3.1620(15)	Au8−Au6^{4}	2.7807(13)
Au8–Au7^{6}	3.1621(15)	Au5–S2	2.378(6)
Au7–S1	2.384(6)	Au8–S1	2.301(8)
Au8–S2	2.308(8)		

Symmetry codes: ^{1}*1-X,1-Y,+Z*; ^{2}*+Z,1-X,1-Y*; ^{3}*1-Y,1-Z,1-X*; ^{4}*+Y,1-Z,+X*; ^{5}*1-Z,1-X,1-Y*; ^{6}*1-Y,1-Z,+X*; ^{7}*1-X,+Y,+Z*; ^{8}*+Y,1-Z,1-X*; ^{9}*1-X,+Y,1-Z*; ^{10}*+X,+Y,1-Z*; ^{11}*+X,1-Y,+Z*; ^{12}*+Z,+X,1-Y*; ^{13}*1-Y,+Z,+X*.

Supplementary Table 3. Bond angles in $Au_{138}(SR)_{48}$

Bond	Angle/°	Bond	Angle/°
Au1^{1}–Au1–Au1^{2}	60.24(3)	Au1^{2}–Au1–Au1^{3}	107.68(4)
Au1^{3}–Au1–Au1^{4}	59.51(6)	Au1^{5}–Au1–Au1^{3}	60.0
Au1^{1}–Au1–Au1^{3}	108.26(3)	Au1^{2}–Au1–Au2^{5}	92.10(5)
Au1^{2}–Au1–Au1^{5}	108.121(14)	Au1^{1}–Au1–Au2^{5}	61.32(3)
Au1^{2}–Au1–Au1^{4}	59.998(1)	Au1^{4}–Au1–Au3	90.92(2)
Au1^{3}–Au1–Au2^{5}	149.39(4)	Au1^{3}–Au1–Au3^{3}	60.79(3)
Au1^{2}–Au1–Au3^{6}	90.77(3)	Au1^{5}–Au1–Au3	150.31(6)
Au1^{1}–Au1–Au3^{6}	148.31(2)	Au1^{3}–Au1–Au3^{7}	90.92(2)
Au1^{2}–Au1–Au3	61.04(3)	Au1^{1}–Au1–Au3	92.38(3)
Au1^{3}–Au1–Au3	147.71(6)	Au1^{2}–Au1–Au3^{7}	150.31(6)
Au1^{4}–Au1–Au3^{6}	60.79(3)	Au1^{4}–Au1–Au3^{3}	91.80(6)
Au1^{1}–Au1–Au3^{3}	148.31(2)	Au1^{4}–Au1–Au3^{7}	147.71(6)
Au1^{3}–Au1–Au3^{6}	91.80(6)	Au1^{1}–Au1–Au4^{3}	122.79(3)
Au1^{3}–Au1–Au4^{3}	120.73(4)	Au1^{4}–Au1–Au4^{3}	120.73(4)
Au1^{5}–Au1–Au4^{3}	122.15(2)	Au1^{2}–Au1–Au4^{3}	122.15(2)
Au1^{3}–Au2–Au3^{8}	58.42(3)	Au1^{4}–Au2–Au1^{3}	57.37(5)
Au1^{3}–Au2–Au3^{4}	87.69(4)	Au1^{4}–Au2–Au3^{3}	87.69(4)
Au1^{4}–Au2–Au3^{6}	58.42(3)	Au1^{3}–Au2–Au3^{6}	87.69(4)
Au1^{3}–Au2–Au3^{3}	58.42(3)	Au1^{4}–Au2–Au3^{4}	58.42(3)
Au1^{3}–Au2–Au4^{2}	117.87(6)	Au1^{4}–Au2–Au3^{8}	87.69(4)
Au1^{4}–Au2–Au4^{2}	60.50(4)	Au1^{3}–Au2–Au4^{5}	60.50(4)
Au1–Au3–Au1^{2}	58.17(4)	Au1^{2}–Au3–Au2^{5}	88.41(4)
Au1–Au3–Au2^{5}	59.03(4)	Au1–Au3–Au3^{6}	60.07(3)
Au1^{2}–Au3–Au3^{11}	58.45(2)	Au1–Au3–Au3^{11}	87.63(3)
Au1–Au3–Au3^{2}	89.23(3)	Au12–Au3–Au3^{2}	59.83(3)
Au1^{2}–Au3–Au3^{6}	89.09(2)	Au1^{2}–Au3–Au4	61.48(4)
Au1–Au3–Au4	119.62(4)	Au1–Au3–Au4^{3}	61.00(3)
Au1^{2}–Au3–Au4^{3}	119.17(4)	Au1^{2}–Au3–Au5^{2}	141.47(4)
Au1–Au3–Au5^{2}	104.49(4)	Au1^{2}–Au3–Au7^{2}	107.21(4)
Au1–Au3–Au7^{2}	143.00(4)	Au1^{2}–Au4–Au2^{4}	58.04(4)
Au1^{2}–Au4–Au3^{2}	57.89(3)	Au1^{2}–Au4–Au3^{12}	57.89(3)
Au1^{4}–Au2–Au4^{5}	117.87(6)	Au2–Au5–Au3^{6}	61.91(3)
Au2–Au5–Au4^{2}	64.05(3)	Au2–Au5–Au5^{10}	59.10(2)
Au2–Au5–Au5^{7}	58.45(2)	Au2–Au5–Au6^{3}	85.12(3)
Au2–Au5–Au7^{6}	105.91(4)	Au2–Au5–Au8	136.41(4)
Au2^{5}–Au3–Au3^{11}	59.770(17)	Au2^{5}–Au3–Au5^{2}	55.26(4)
Au2^{5}–Au1–Au4^{3}	61.47(4)	Au3^{6}–Au2–Au3^{4}	106.64(4)
Au3^{6}–Au2–Au3^{3}	60.46(3)	Au3^{8}–Au2–Au3^{3}	106.64(4)
Au3^{3}–Au2–Au3^{4}	142.50(7)	Au3^{8}–Au2–Au3^{4}	60.46(3)
Au3^{6}–Au2–Au3^{8}	142.50(7)	Au3^{3}–Au2–Au4^{5}	59.469(19)
Au3^{4}–Au2–Au4^{5}	119.93(3)	Au3^{3}–Au2–Au4^{2}	119.93(3)
Au3^{8}–Au2–Au4^{5}	59.469(19)	Au3–Au1–Au2^{5}	62.56(3)
Au3–Au4–Au3^{2}	58.54(4)	Au3^{3}–Au1–Au2^{5}	111.53(4)
Au3^{7}–Au1–Au2^{5}	62.56(3)	Au3–Au1–Au3^{3}	112.08(4)
Au3^{6}–Au1–Au2^{5}	111.53(4)	Au3^{7}–Au1–Au3^{3}	60.11(4)

$Au3^{3}$–Au1–$Au3^{6}$	63.10(4)	Au3–Au1–$Au3^{7}$	113.33(5)
$Au3^{7}$–Au1–$Au3^{6}$	112.08(4)	$Au3^{7}$–Au1–$Au4^{3}$	61.11(3)
Au3–Au1–$Au3^{6}$	60.11(4)	$Au3^{3}$–Au1–$Au4^{3}$	59.97(3)
$Au3^{6}$–Au1–$Au4^{3}$	59.97(3)	Au3–Au4–$Au3^{12}$	108.15(5)
Au3–Au1–$Au4^{3}$	61.11(3)	$Au3^{11}$–Au4–$Au3^{2}$	108.16(5)
$Au3^{2}$–Au4–$Au3^{12}$	107.86(5)	$Au3^{11}$–Au4–$Au7^{2}$	118.66(5)
$Au3^{11}$–Au4–$Au3^{12}$	58.54(4)	$Au3^{11}$–Au4–$Au7^{12}$	60.89(3)
Au3–Au4–$Au7^{12}$	118.66(5)	$Au3^{6}$–Au5–Au8	80.17(3)
Au3–Au4–$Au7^{2}$	60.90(3)	$Au3^{6}$–Au5–$Au7^{6}$	52.45(3)
$Au3^{11}$–Au6–Au4	63.18(4)	Au3–Au6–Au4	63.18(4)
$Au3^{11}$–Au6–Au3	66.53(5)	Au3–Au6–$Au5^{12}$	95.39(5)
Au3–Au6–$Au8^{2}$	92.16(3)	$Au3^{11}$–Au6–$Au8^{2}$	157.42(5)
Au3–Au6–$Au8^{12}$	157.41(5)	$Au3^{11}$–Au6–$Au8^{12}$	92.16(3)
Au3–Au7–$Au4^{3}$	62.70(3)	Au3–Au7–$Au3^{6}$	60.15(4)
Au3–Au7–$Au5^{2}$	59.52(3)	$Au3^{6}$–Au7–$Au5^{2}$	104.42(4)
Au3–Au7–$Au7^{2}$	63.17(3)	Au3–Au7–$Au7^{6}$	91.75(3)
Au3–Au7–Au8	139.41(4)	$Au3^{6}$–Au7–$Au8^{2}$	142.73(4)
Au3–Au7–$Au8^{2}$	83.87(4)	$Au3^{6}$–Au7–Au8	79.30(4)
$Au3^{2}$–Au3–$Au2^{5}$	144.75(3)	$Au3^{6}$–Au3–$Au2^{5}$	108.62(4)
$Au3^{6}$–Au3–$Au3^{2}$	60.0	$Au3^{6}$–Au3–$Au3^{11}$	144.020(5)
$Au3^{6}$–Au3–$Au4^{3}$	59.92(4)	$Au3^{2}$–Au3–$Au3^{11}$	108.06(2)
$Au3^{6}$–Au3–Au4	121.54(4)	$Au3^{2}$–Au3–$Au4^{3}$	119.92(4)
$Au3^{2}$–Au3–$Au5^{2}$	158.64(2)	$Au3^{2}$–Au3–Au4	61.54(4)
$Au3^{6}$–Au3–$Au5^{2}$	112.71(5)	$Au3^{11}$–Au3–$Au5^{2}$	89.09(2)
$Au3^{2}$–Au3–$Au7^{2}$	56.69(3)	$Au3^{6}$–Au3–$Au7^{2}$	88.00(3)
Au3–Au4–$Au2^{4}$	107.48(5)	$Au3^{11}$–Au4–$Au1^{2}$	58.55(3)
$Au3^{11}$–Au4–Au3	62.07(4)	$Au3^{12}$–Au4–$Au2^{4}$	60.24(3)
$Au4^{2}$–Au5–$Au3^{6}$	60.64(3)	$Au4^{2}$–Au5–$Au5^{7}$	122.49(3)
$Au4^{2}$–Au5–$Au5^{10}$	59.93(2)	$Au4^{2}$–Au5–$Au6^{3}$	115.25(4)
$Au4^{2}$–Au5–$Au7^{6}$	56.84(3)	$Au4^{2}$–Au5–Au8	116.30(4)
$Au4^{2}$–Au2–$Au4^{5}$	178.37(8)	Au4–Au6–$Au5^{2}$	126.76(4)
Au4–Au6–$Au5^{12}$	126.76(4)	$Au4^{3}$–Au7–$Au3^{6}$	59.51(3)
$Au4^{3}$–Au7–$Au5^{2}$	54.84(3)	$Au4^{3}$–Au7–Au8	99.59(4)
$Au4^{3}$–Au7–$Au8^{2}$	114.29(5)	Au4–Au3–$Au2^{5}$	118.70(3)
$Au4^{3}$–Au3–$Au2^{5}$	60.29(3)	Au4–Au3–$Au3^{11}$	58.96(2)
$Au4^{3}$–Au3–$Au3^{11}$	120.05(3)	Au4–Au3–$Au4^{3}$	178.53(4)
$Au4^{3}$–Au3–$Au5^{2}$	57.00(3)	Au4–Au3–$Au5^{2}$	121.61(4)
Au4–Au3–$Au7^{2}$	59.60(3)	$Au5^{13}$–Au4–$Au1^{2}$	106.23(4)
$Au5^{13}$–Au4–$Au2^{4}$	56.85(4)	$Au5^{6}$–Au4–$Au1^{2}$	106.23(4)
$Au5^{6}$–Au4–$Au2^{4}$	56.85(4)	$Au5^{6}$–Au4–$Au3^{12}$	110.25(5)
$Au5^{13}$–Au4–$Au3^{11}$	116.37(3)	$Au5^{13}$–Au4–$Au3^{12}$	62.36(3)
$Au5^{6}$–Au4–Au3	116.37(3)	$Au5^{6}$–Au4–$Au3^{2}$	62.36(3)
$Au5^{13}$–Au4–Au3	163.91(5)	$Au5^{6}$–Au4–$Au3^{11}$	163.91(5)
$Au5^{13}$–Au4–$Au3^{2}$	110.25(5)	$Au5^{13}$–Au4–$Au5^{6}$	60.14(5)
$Au5^{13}$–Au4–$Au7^{12}$	68.32(3)	$Au5^{6}$–Au4–$Au7^{12}$	124.72(5)
$Au5^{13}$–Au4–$Au7^{2}$	124.72(5)	$Au5^{6}$–Au4–$Au7^{2}$	68.32(3)
$Au5^{10}$–Au5–$Au3^{6}$	108.72(2)	$Au5^{7}$–Au5–$Au3^{6}$	90.91(2)
$Au5^{10}$–Au5–$Au5^{7}$	90.0	$Au5^{10}$–Au5–$Au6^{3}$	142.92(3)
$Au5^{7}$–Au5–$Au6^{3}$	60.58(2)	$Au5^{10}$–Au5–$Au7^{6}$	113.78(3)

Au5^{7}–Au5–Au7^{6}	140.38(2)	Au5^{7}–Au5–Au8	104.70(3)
Au5^{10}–Au5–Au8	162.92(3)	Au5^{9}–Au2–Au1^{4}	147.97(3)
Au5^{9}–Au2–Au1^{3}	110.21(3)	Au5^{10}–Au2–Au1^{4}	110.21(3)
Au5^{10}–Au2–Au1^{3}	147.97(3)	Au5–Au2–Au1^{3}	147.97(3)
Au5^{7}–Au2–Au1^{4}	147.97(3)	Au5^{7}–Au2–Au3^{4}	153.37(5)
Au5^{9}–Au2–Au3^{6}	153.37(5)	Au5^{10}–Au2–Au3^{8}	94.03(3)
Au5^{10}–Au2–Au3^{4}	62.83(3)	Au5–Au2–Au3^{8}	153.37(5)
Au5^{10}–Au2–Au3^{6}	111.53(3)	Au5^{10}–Au2–Au3	153.37(5)
Au5–Au2–Au3^{6}	62.83(3)	Au5–Au2–Au3^{3}	94.03(3)
Au5^{9}–Au2–Au3^{4}	94.03(3)	Au5^{7}–Au2–Au3	94.03(3)
Au5^{7}–Au2–Au3^{3}	62.83(3)	Au5–Au2–Au4^{5}	122.20(5)
Au5^{7}−Au2−Au4^{2}	122.20(5)	Au5−Au2−Au4^{2}	59.10(3)
Au5^{10}–Au2–Au4^{2}	59.10(3)	Au5^{2}–Au7–Au8	141.46(4)
Au5^{9}–Au2–Au4^{2}	122.20(5)	Au5–Au8–Au7^{6}	61.04(3)
Au5^{10}–Au2–Au4^{5}	122.20(5)	Au5^{10}–Au2–Au5	61.80(5)
Au5^{9}–Au2–Au5	94.31(6)	Au5–Au8–Au7	93.86(4)
Au5^{9}–Au2–Au5	61.80(5)	Au5^{7}–Au2–Au5^{10}	94.31(6)
Au5^{7}–Au2–Au5	63.11(5)	Au5^{9}–Au2–Au5^{10}	63.11(5)
Au6–Au4–Au2^{4}	161.07(6)	Au6–Au4–Au1^{2}	103.03(5)
Au6–Au4–Au3^{2}	111.51(4)	Au6–Au4–Au3^{12}	111.51(4)
Au6–Au4–Au3	57.01(3)	Au6–Au4–Au3^{11}	57.01(3)
Au6–Au4–Au5^{6}	137.30(4)	Au6–Au4–Au5^{13}	137.30(4)
Au6–Au4–Au7^{2}	74.19(3)	Au6–Au4–Au7^{12}	74.19(3)
Au6^{3}–Au5–Au7^{6}	83.34(3)	Au6^{3}–Au5–Au3^{6}	54.61(3)
Au6^{3}–Au8–Au5	58.83(4)	Au6^{3}–Au5–Au8	54.10(3)
Au6^{3}–Au8–Au7^{6}	86.81(4)	Au6^{3}–Au8–Au7	68.74(4)
Au6–Au3–Au1	140.04(4)	Au6–Au3–Au1^{2}	107.51(4)
Au6–Au3–Au3^{11}	56.73(2)	Au6–Au3–Au2^{5}	85.40(4)
Au6–Au3–Au3^{2}	116.84(5)	Au6–Au3–Au3^{6}	158.85(2)
Au6–Au3–Au4^{3}	118.80(5)	Au6–Au3–Au4	59.81(4)
Au6–Au3–Au7	97.06(4)	Au6–Au3–Au5^{2}	61.80(4)
Au6–Au3–Au7^{2}	74.80(4)	Au7^{12}–Au4–Au1^{2}	105.95(3)
Au7^{2}–Au4–Au1^{2}	105.95(3)	Au7^{2}–Au4–Au2^{4}	109.19(3)
Au7^{12}–Au4–Au2^{4}	109.19(3)	Au7^{12}–Au4–Au3^{2}	163.35(5)
Au7^{2}–Au4–Au3^{12}	163.35(5)	Au7^{12}–Au4–Au3^{12}	56.19(3)
Au7^{2}–Au4–Au3^{2}	56.19(3)	Au7^{6}–Au7–Au3^{6}	56.71(3)
Au7^{2}–Au7–Au3^{6}	88.03(3)	Au7^{2}–Au7–Au4^{3}	125.45(3)
Au7^{6}–Au7–Au4^{3}	115.68(4)	Au7^{2}–Au7–Au5^{2}	101.37(5)
Au7^{6}–Au7–Au5^{2}	151.24(3)	Au7^{2}–Au7–Au7^{6}	60.0
Au7^{2}–Au7–Au8	117.16(4)	Au7^{6}–Au7–Au8^{2}	120.45(4)
Au7^{6}–Au7–Au8	61.89(4)	Au7^{2}–Au7–Au8^{2}	65.51(4)
Au7^{6}–Au8–Au7	52.60(4)	Au7–Au3–Au1	112.75(4)
Au7–Au3–Au1^{2}	148.48(4)	Au7^{2}–Au3–Au2^{5}	157.63(5)
Au7–Au3–Au2^{5}	113.47(4)	Au7–Au3–Au3^{11}	152.10(3)
Au7–Au3–Au3^{6}	63.15(3)	Au7^{2}–Au3–Au3^{11}	114.61(3)
Au7–Au3–Au3^{2}	91.72(3)	Au7–Au3–Au4^{3}	61.11(3)
Au7^{2}–Au3–Au4^{3}	120.85(4)	Au7–Au3–Au4	119.14(4)
Au7–Au3–Au5^{2}	68.03(3)	Au7^{2}–Au3–Au5^{2}	105.01(4)
Au7–Au3–Au7^{2}	60.12(4)	Au7^{12}–Au4–Au7^{2}	139.08(6)

Au8–Au5–Au7^{6}	59.49(3)	Au8^{2}–Au6–Au4	115.04(4)
Au8^{12}–Au6–Au4	115.04(4)	Au8^{12}–Au6–Au5^{2}	113.80(5)
Au8^{2}–Au6–Au5^{12}	113.80(5)	Au8^{2}–Au6–Au5^{2}	67.08(4)
Au8^{2}–Au7–Au5^{2}	59.47(3)	Au8^{2}–Au7–Au8	135.33(5)
Au8^{12}–Au6–Au5^{12}	67.08(4)	Au8^{12}–Au6–Au8^{2}	107.66(6)

Symmetry codes: ^{1}*1-X,1-Y,+Z*; ^{2}*+Z,1-X,1-Y*; ^{3}*1-Y,1-Z,1-X*; ^{4}*+Y,1-Z,+X*; ^{5}*1-Z,1-X,1-Y*; ^{6}*1-Y,1-Z,+X*; ^{7}*1-X,+Y,+Z*; ^{8}*+Y,1-Z,1-X*; ^{9}*1-X,+Y,1-Z*; ^{10}*+X,+Y,1-Z*; ^{11}*+X,1-Y,+Z*; ^{12}*+Z,+X,1-Y*; ^{13}*1-Y,+Z,+X*.

Supplementary Table 4. Analysis of selected excited states with largest oscillator strengths contributing to the peaks in theoretical UV-vis spectra

Excited state	Wavelength (nm) Energy (eV)	Osc. str.	Transition (Occ → Vir)	Orbital energy (eV)	ΔE[a] (eV)	Transition coefficient	Orbital decomposition			
							Au (6sp)	Au (5d)	S	C, H
**Peak 3 (540 nm)**										
2511	517 (2.40)	0.036	2986[b]	-5.19	2.40	0.29	0.13	0.39	0.20	0.28
			3075	-2.79			0.56	0.24	0.12	0.08
			2986	-5.19	2.40	0.25	0.13	0.39	0.20	0.28
			3076	-2.79			0.56	0.24	0.12	0.08
			3061	-3.75	2.39	0.16	0.44	0.39	0.13	0.04
			3111	-1.36			0.57	0.12	0.12	0.19
2028	548 (2.26)	0.020	2964	-5.37	2.26	0.37	0.08	0.29	0.13	0.50
			3069	-3.11			0.56	0.27	0.13	0.04
			3053	-4.00	2.27	0.32	0.40	0.32	0.20	0.08
			3106	-1.73			0.65	0.13	0.08	0.14
			2964	-5.37	2.26	0.09	0.08	0.29	0.13	0.50
			3070	-3.11			0.57	0.24	0.13	0.06
2121	539 (2.30)	0.018	2920	-5.64	2.30	0.50	0.03	0.42	0.03	0.52
			3065	-3.35			0.50	0.30	0.14	0.06
			3033	-4.78	2.30	0.19	0.20	0.35	0.25	0.20
			3081	-2.48			0.63	0.02	0.12	0.23
			3034	-4.77	2.29	0.15	0.21	0.35	0.24	0.20
			3083	-2.48			0.57	0.20	0.14	0.09
2128	540 (2.30)	0.017	2919	-5.65	2.30	0.70	0.03	0.42	0.02	0.53
			3064	-3.35			0.76	0.10	0.08	0.06
			3029	-4.79	2.31	0.15	0.20	0.32	0.29	0.19
			3082	-2.48			0.57	0.20	0.14	0.09
			2907	-5.69	2.34	0.12	0.04	0.49	0.05	0.42
			3063	-3.35			0.60	0.23	0.14	0.03

Peak 2 (430 nm)

5418	431 (2.87)	0.017	3024	-4.83	2.88	0.34	0.10	0.31	0.28	0.30
			3098	-1.95			0.60	0.19	0.12	0.09
			2950	-5.50	2.88	0.17	0.04	0.51	0.04	0.40
			3078	-2.62			0.54	0.25	0.17	0.04
			2984	-5.21	2.88	0.12	0.10	0.36	0.23	0.31
			3089	-2.33			0.65	0.17	0.09	0.09
4696	445 (2.78)	0.017	3011	-4.96	2.79	0.15	0.13	0.41	0.23	0.24
			3094	-2.17			0.67	0.13	0.11	0.09
			2784	-6.18	2.79	0.14	0.05	0.76	0.06	0.13
			3063	-3.39			0.60	0.23	0.14	0.04
			3057	-3.92	2.79	0.12	0.44	0.37	0.14	0.05
			3126	-1.13			0.11	0.01	0.01	0.86
4401	452 (2.74)	0.015	3058	-3.88	2.75	0.81	0.44	0.37	0.14	0.05
			3126	-1.13			0.11	0.01	0.01	0.86
			2979	-5.24	2.76	0.06	0.09	0.28	0.25	0.38
			3082	-2.48			0.57	0.20	0.14	0.09
			3017	-4.92	2.75	0.05	0.11	0.35	0.26	0.28
			3093	-2.17			0.67	0.13	0.11	0.09
5788	424 (2.92)	0.014	2820	-6.04	2.93	0.22	0.03	0.73	0.04	0.20
			3067	-3.11			0.56	0.27	0.14	0.03
			2897	-5.72	2.93	0.13	0.06	0.49	0.07	0.38
			3076	-2.79			0.56	0.24	0.12	0.09
			2898	-5.72	2.92	0.11	0.07	0.67	0.07	0.18
			3076	-2.79			0.56	0.24	0.12	0.09

Peak 1 (360 nm)

11647	358 (3.46)	0.029	3047	-4.21	3.46	0.29	0.28	0.40	0.21	0.11
			3172	-0.74			0.16	0.02	0.05	0.77
			2871	-5.82	3.46	0.17	0.04	0.71	0.04	0.21
			3087	-2.36			0.64	0.17	0.09	0.10
			2881	-5.79	3.46	0.12	0.03	0.33	0.03	0.61
			3090	-2.33			0.77	0.10	0.07	0.06
11661	358 (3.46)	0.020	2867	-5.86	3.46	0.16	0.02	0.73	0.05	0.20
			3085	-2.39			0.70	0.13	0.09	0.08
			3052	-4.03	3.47	0.11	0.40	0.32	0.20	0.08
			3205	-0.56			0.14	0.02	0.04	0.80
			2881	-5.79	3.46	0.10	0.03	0.33	0.03	0.61
			3090	-2.33			0.77	0.10	0.07	0.06
11929	356 (3.48)	0.015	3032	-4.78	3.49	0.42	0.16	0.32	0.27	0.25
			3114	-1.29			0.53	0.08	0.09	0.30
			2954	-5.45	3.49	0.07	0.02	0.46	0.06	0.46
			3097	-1.96			0.60	0.19	0.12	0.09
			2803	-6.10	3.48	0.06	0.05	0.57	0.05	0.33
			3078	-2.62			0.54	0.25	0.17	0.04
10524	368 (3.37)	0.014	2904	-5.70	3.38	0.48	0.03	0.37	0.05	0.55
			3091	-2.33			0.77	0.10	0.07	0.06
			3051	-4.03	3.37	0.16	0.40	0.32	0.20	0.08
			3189	-0.66			0.08	0.01	0.03	0.88
			2967	-5.34	3.37	0.11	0.11	0.39	0.09	0.41
			3097	-1.96			0.60	0.19	0.12	0.09

[a]Energy difference between the occupied and virtual level
[b]For MOs, 3062 and 3063 represent the HOMO and LUMO levels respectively. All of the other MO numbers are consistent with this representation.

3. Supplementary references

1. Yan, N. *et al*. Unraveling the long-pursued Au_{144} structure by x-ray crystallography. *Sci. Adv.* **4**, eaat7259 (2018).

4. CheckCIF report and explanations

checkCIF/PLATON report

Structure factors have been supplied for datablock(s) ca_b

THIS REPORT IS FOR GUIDANCE ONLY. IF USED AS PART OF A REVIEW PROCEDURE FOR PUBLICATION, IT SHOULD NOT REPLACE THE EXPERTISE OF AN EXPERIENCED CRYSTALLOGRAPHIC REFEREE.

No syntax errors found. CIF dictionary Interpreting this report

Datablock: ca_b

```
Bond precision:   C-C = 0.0525 A           Wavelength=1.54178

Cell:              a=52.7982(8)      b=52.7982(8)      c=52.7982(8)
                   alpha=90          beta=90           gamma=90
Temperature:       173 K
```

	Calculated	Reported
Volume	147183(7)	147183(7)
Space group	F m -3 c	F m -3 c
Hall group	-F 4c 2 3	-F 4c 2 3
Moiety formula	C384 H432 Au138 S48 [+ solvent]	4(C384 H432 Au138 S48)
Sum formula	C384 H432 Au138 S48 [+ solvent]	C1536 H0.50 Au552 S192
Mr	33768.05	133328.89
Dx,g cm-3	3.048	3.008
Z	8	2
Mu (mm-1)	51.667	51.665
F000	115248.0	111793.0
F000’	110813.52	
h,k,lmax	62,62,62	62,62,62
Nref	5675	5676
Tmin,Tmax	0.811,0.772	0.413,0.753
Tmin’	0.736	

```
Correction method= # Reported T Limits: Tmin=0.413 Tmax=0.753
AbsCorr = NONE

Data completeness= 1.000            Theta(max)= 66.886
```

R(reflections)= 0.0567(4813) wR2(reflections)= 0.1685(5676)

S = 1.223 Npar= 287

The following ALERTS were generated. Each ALERT has the format
test-name_ALERT_alert-type_alert-level.
Click on the hyperlinks for more details of the test.

Alert level A

PLAT972_ALERT_2_A Check Calcd Resid. Dens. 2.63Ang From Au1 -3.85 eA-3

Author Response: The unexpected residual density is due to the poor crystal quality. Data re-collection with several crystal samples gave a similar result. Reintegrating the diffraction images with larger box sizes did not improve the situation. Other characterizations consistently support the current structural model.

PLAT974_ALERT_2_A Check Calcd Negative Resid. Density on Au1 -3.32 eA-3

Author Response: The unexpected residual density is due to the poor crystal quality. Data re-collection with several crystal samples gave a similar result. Reintegrating the diffraction images with larger box sizes did not improve the situation. Other characterizations consistently support the current structural model.

PLAT974_ALERT_2_A Check Calcd Negative Resid. Density on Au4 -2.56 eA-3

Author Response: The unexpected residual density is due to the poor crystal quality. Data re-collection with several crystal samples gave a similar result. Reintegrating the diffraction images with larger box sizes did not improve the situation. Other characterizations consistently support the current structural model.

PLAT974_ALERT_2_A Check Calcd Negative Resid. Density on Au4 -2.56 eA-3

Author Response: The unexpected residual density is due to the poor crystal quality. Data re-collection with several crystal samples gave a similar result. Reintegrating the diffraction images with larger box sizes did not improve the situation. Other characterizations consistently support the current structural model.

PLAT974_ALERT_2_A Check Calcd Negative Resid. Density on Au4 -2.56 eA-3

Author Response: The unexpected residual density is due to the poor crystal quality. Data re-collection with several crystal samples gave a similar result. Reintegrating the diffraction images with larger box sizes did not improve the situation. Other characterizations consistently support the current structural model.

Alert level B

```
PLAT342_ALERT_3_B Low Bond Precision on  C-C Bonds ..............     0.0525 Ang.
```

Author Response: The unexpected residual density is due to the poor crystal quality. Data re-collection with several crystal samples gave a similar result. Reintegrating the diffraction images with larger box sizes did not improve the situation. Other characterizations consistently support the current structural model.

```
PLAT919_ALERT_3_B Reflection # Likely Affected by the Beamstop ...          1 Check
```

Author Response: The unexpected residual density is due to the poor crystal quality. Data re-collection with several crystal samples gave a similar result. Reintegrating the diffraction images with larger box sizes did not improve the situation. Other characterizations consistently support the current structural model.

```
PLAT934_ALERT_3_B Number of (Iobs-Icalc)/Sigma(W) > 10 Outliers ..          3 Check
```

Author Response: The unexpected residual density is due to the poor crystal quality. Data re-collection with several crystal samples gave a similar result. Reintegrating the diffraction images with larger box sizes did not improve the situation. Other characterizations consistently support the current structural model.

```
PLAT972_ALERT_2_B Check Calcd Resid. Dens.  0.99Ang From Au1           -3.31 eA-3
```

Author Response: The unexpected residual density is due to the poor crystal quality. Data re-collection with several crystal samples gave a similar result. Reintegrating the diffraction images with larger box sizes did not improve the situation. Other characterizations consistently support the current structural model.

```
PLAT972_ALERT_2_B Check Calcd Resid. Dens.  1.36Ang From Au1           -3.26 eA-3
```

Author Response: The unexpected residual density is due to the poor crystal quality. Data re-collection with several crystal samples gave a similar result. Reintegrating the diffraction images with larger box sizes did not improve the situation. Other characterizations consistently support the current structural model.

```
PLAT972_ALERT_2_B Check Calcd Resid. Dens.  1.66Ang From Au1           -3.08 eA-3
```

Author Response: The unexpected residual density is due to the poor crystal quality. Data re-collection with several crystal samples gave a similar result. Reintegrating the diffraction images with larger box sizes did not improve the situation. Other characterizations consistently support the current structural model.

```
PLAT972_ALERT_2_B Check Calcd Resid. Dens.  0.93Ang From Au3           -3.00 eA-3
```

Author Response: The unexpected residual density is due to the poor crystal quality. Data re-collection with several crystal samples gave a similar result. Reintegrating the diffraction images with larger box sizes did not improve the situation. Other characterizations consistently support the current structural model.

```
PLAT972_ALERT_2_B Check Calcd Resid. Dens.  0.85Ang From Au1           -2.98 eA-3
```

Author Response: The unexpected residual density is due to the poor crystal quality. Data re-collection with several crystal samples gave a similar result. Reintegrating the diffraction images with larger box sizes did not improve the situation. Other characterizations consistently support the current structural model.

```
PLAT972_ALERT_2_B Check Calcd Resid. Dens.  1.10Ang From Au3           -2.97 eA-3
```

Author Response: The unexpected residual density is due to the poor crystal quality. Data re-collection with several crystal samples gave a similar result. Reintegrating the diffraction images with larger box sizes did not improve the situation. Other characterizations consistently support the current structural model.

```
PLAT972_ALERT_2_B Check Calcd Resid. Dens.  1.10Ang From Au3           -2.88 eA-3
```

Author Response: The unexpected residual density is due to the poor crystal quality. Data re-collection with several crystal samples gave a similar result. Reintegrating the diffraction images with larger box sizes did not improve the situation. Other characterizations consistently support the current structural model.

PLAT972_ALERT_2_B Check Calcd Resid. Dens. 1.49Ang From Au1 -2.87 eA-3

Author Response: The unexpected residual density is due to the poor crystal quality. Data re-collection with several crystal samples gave a similar result. Reintegrating the diffraction images with larger box sizes did not improve the situation. Other characterizations consistently support the current structural model.

PLAT972_ALERT_2_B Check Calcd Resid. Dens. 1.49Ang From Au1 -2.77 eA-3

Author Response: The unexpected residual density is due to the poor crystal quality. Data re-collection with several crystal samples gave a similar result. Reintegrating the diffraction images with larger box sizes did not improve the situation. Other characterizations consistently support the current structural model.

PLAT972_ALERT_2_B Check Calcd Resid. Dens. 1.70Ang From Au3 -2.76 eA-3

Author Response: The unexpected residual density is due to the poor crystal quality. Data re-collection with several crystal samples gave a similar result. Reintegrating the diffraction images with larger box sizes did not improve the situation. Other characterizations consistently support the current structural model.

PLAT972_ALERT_2_B Check Calcd Resid. Dens. 1.16Ang From Au4 -2.74 eA-3

Author Response: The unexpected residual density is due to the poor crystal quality. Data re-collection with several crystal samples gave a similar result. Reintegrating the diffraction images with larger box sizes did not improve the situation. Other characterizations consistently support the current structural model.

PLAT972_ALERT_2_B Check Calcd Resid. Dens. 1.03Ang From Au6 -2.70 eA-3

Author Response: The unexpected residual density is due to the poor crystal quality. Data re-collection with several crystal samples gave a similar result. Reintegrating the diffraction images with larger box sizes did not improve the situation. Other characterizations consistently support the current structural model.

PLAT972_ALERT_2_B Check Calcd Resid. Dens. 1.03Ang From Au6 -2.68 eA-3

Author Response: The unexpected residual density is due to the poor crystal quality. Data re-collection with several crystal samples gave a similar result. Reintegrating the diffraction images with larger box sizes did not improve the situation. Other characterizations consistently support the current structural model.

PLAT972_ALERT_2_B Check Calcd Resid. Dens. 1.11Ang From Au3 -2.65 eA-3

Author Response: The unexpected residual density is due to the poor crystal quality. Data re-collection with several crystal samples gave a similar result. Reintegrating the diffraction images with larger box sizes did not improve the situation. Other characterizations consistently support the current structural model.

PLAT972_ALERT_2_B Check Calcd Resid. Dens. 0.99Ang From Au1 -2.64 eA-3

Author Response: The unexpected residual density is due to the poor crystal quality. Data re-collection with several crystal samples gave a similar result. Reintegrating the diffraction images with larger box sizes did not improve the situation. Other characterizations consistently support the current structural model.

PLAT972_ALERT_2_B Check Calcd Resid. Dens. 0.99Ang From Au1 -2.64 eA-3

Author Response: The unexpected residual density is due to the poor crystal quality. Data re-collection with several crystal samples gave a similar result. Reintegrating the diffraction images with larger box sizes did not improve the situation. Other characterizations consistently support the current structural model.

PLAT972_ALERT_2_B Check Calcd Resid. Dens. 0.99Ang From Au1 -2.64 eA-3

Author Response: The unexpected residual density is due to the poor crystal quality. Data re-collection with several crystal samples gave a similar result. Reintegrating the diffraction images with larger box sizes did not improve the situation. Other characterizations consistently support the current structural model.

PLAT972_ALERT_2_B Check Calcd Resid. Dens. 1.17Ang From Au3   -2.62 eA-3

Author Response: The unexpected residual density is due to the poor crystal quality. Data re-collection with several crystal samples gave a similar result. Reintegrating the diffraction images with larger box sizes did not improve the situation. Other characterizations consistently support the current structural model.

PLAT972_ALERT_2_B Check Calcd Resid. Dens. 1.19Ang From Au1   -2.62 eA-3

Author Response: The unexpected residual density is due to the poor crystal quality. Data re-collection with several crystal samples gave a similar result. Reintegrating the diffraction images with larger box sizes did not improve the situation. Other characterizations consistently support the current structural model.

PLAT972_ALERT_2_B Check Calcd Resid. Dens. 1.19Ang From Au1   -2.62 eA-3

Author Response: The unexpected residual density is due to the poor crystal quality. Data re-collection with several crystal samples gave a similar result. Reintegrating the diffraction images with larger box sizes did not improve the situation. Other characterizations consistently support the current structural model.

Alert level C

CHEMW01_ALERT_1_C The difference between the given and expected weight for
compound is greater 1 mass unit. Check that all hydrogen
atoms have been taken into account.
RINTA01_ALERT_3_C The value of Rint is greater than 0.12
Rint given   0.167
PLAT018_ALERT_1_C _diffrn_measured_fraction_theta_max .NE. *_full   ! Check
PLAT906_ALERT_3_C Large K Value in the Analysis of Variance ......   7.116 Check
PLAT971_ALERT_2_C Check Calcd Resid. Dens. 2.83Ang From C8A   2.14 eA-3
PLAT971_ALERT_2_C Check Calcd Resid. Dens. 1.73Ang From C8A   2.07 eA-3
PLAT971_ALERT_2_C Check Calcd Resid. Dens. 1.73Ang From C8A   2.03 eA-3
PLAT971_ALERT_2_C Check Calcd Resid. Dens. 1.73Ang From C8A   2.03 eA-3
PLAT971_ALERT_2_C Check Calcd Resid. Dens. 1.73Ang From C8A   1.99 eA-3
PLAT971_ALERT_2_C Check Calcd Resid. Dens. 1.73Ang From C8A   1.98 eA-3
PLAT971_ALERT_2_C Check Calcd Resid. Dens. 1.73Ang From C8A   1.95 eA-3
PLAT971_ALERT_2_C Check Calcd Resid. Dens. 1.80Ang From C3A   1.89 eA-3

```
PLAT971_ALERT_2_C Check Calcd Resid. Dens.  1.80Ang From C3A           1.86 eA-3
PLAT971_ALERT_2_C Check Calcd Resid. Dens.  1.80Ang From C3A           1.80 eA-3
PLAT971_ALERT_2_C Check Calcd Resid. Dens.  1.41Ang From C7B           1.77 eA-3
PLAT971_ALERT_2_C Check Calcd Resid. Dens.  1.41Ang From C7B           1.77 eA-3
PLAT971_ALERT_2_C Check Calcd Resid. Dens.  2.95Ang From C8A           1.74 eA-3
PLAT971_ALERT_2_C Check Calcd Resid. Dens.  2.95Ang From C8A           1.74 eA-3
PLAT971_ALERT_2_C Check Calcd Resid. Dens.  1.58Ang From C4A           1.72 eA-3
PLAT971_ALERT_2_C Check Calcd Resid. Dens.  3.18Ang From C7A           1.70 eA-3
PLAT971_ALERT_2_C Check Calcd Resid. Dens.  2.17Ang From C7A           1.67 eA-3
PLAT971_ALERT_2_C Check Calcd Resid. Dens.  2.17Ang From C7A           1.67 eA-3
PLAT971_ALERT_2_C Check Calcd Resid. Dens.  2.57Ang From C2B           1.66 eA-3
PLAT971_ALERT_2_C Check Calcd Resid. Dens.  2.57Ang From C2B           1.62 eA-3
PLAT971_ALERT_2_C Check Calcd Resid. Dens.  2.57Ang From C2B           1.62 eA-3
PLAT971_ALERT_2_C Check Calcd Resid. Dens.  2.80Ang From C8A           1.61 eA-3
PLAT971_ALERT_2_C Check Calcd Resid. Dens.  2.80Ang From C8A           1.60 eA-3
PLAT971_ALERT_2_C Check Calcd Resid. Dens.  2.80Ang From C8A           1.59 eA-3
PLAT971_ALERT_2_C Check Calcd Resid. Dens.  2.80Ang From C8A           1.59 eA-3
PLAT977_ALERT_2_C Check Negative Difference Density on H15B      .    -0.39 eA-3
```

Alert level G

```
FORMU01_ALERT_1_G  There is a discrepancy between the atom counts in the
            _chemical_formula_sum and _chemical_formula_moiety. This is
            usually due to the moiety formula being in the wrong format.
            Atom count from _chemical_formula_sum:   C1536 H0.5 Au552 S192
            Atom count from _chemical_formula_moiety:C1536 H1728 Au552 S192
FORMU01_ALERT_2_G  There is a discrepancy between the atom counts in the
            _chemical_formula_sum and the formula from the _atom_site* data.
            Atom count from _chemical_formula_sum:C1536 H0.5 Au552 S192
            Atom count from the _atom_site data:  C1536 H1728 Au552 S192
CELLZ01_ALERT_1_G Difference between formula and atom_site contents detected.
CELLZ01_ALERT_1_G ALERT: Large difference may be due to a
            symmetry error - see SYMMG tests
           From the CIF: _cell_formula_units_Z    2
           From the CIF: _chemical_formula_sum  C1536 H0.50 Au552 S192
           TEST: Compare cell contents of formula and atom_site data

           atom    Z*formula  cif sites diff
           C       3072.00   3072.00    0.00
           H          1.00   3456.00-3455.00
           Au      1104.00   1104.00    0.00
           S        384.00    384.00    0.00
PLAT002_ALERT_2_G Number of Distance or Angle Restraints on AtSite         17 Note
PLAT003_ALERT_2_G Number of Uiso or Uij Restrained non-H Atoms ...         34 Report
PLAT020_ALERT_3_G The Value of Rint is Greater Than 0.12 .........      0.167 Report
PLAT041_ALERT_1_G Calc. and Reported SumFormula    Strings  Differ     Please Check
PLAT045_ALERT_1_G Calculated and Reported Z Differ by a Factor ...          4 Check
PLAT083_ALERT_2_G SHELXL Second Parameter in WGHT  Unusually Large   32898.48 Why ?
PLAT174_ALERT_4_G The CIF-Embedded .res File Contains FLAT Records          1 Report
PLAT176_ALERT_4_G The CIF-Embedded .res File Contains SADI Records         23 Report
PLAT178_ALERT_4_G The CIF-Embedded .res File Contains SIMU Records          1 Report
PLAT186_ALERT_4_G The CIF-Embedded .res File Contains ISOR Records          1 Report
PLAT187_ALERT_4_G The CIF-Embedded .res File Contains RIGU Records          2 Report
PLAT190_ALERT_3_G A Non-default RIGU Restraint Value for First Par     0.0010 Report
PLAT190_ALERT_3_G A Non-default RIGU Restraint Value for SecondPar     0.0010 Report
PLAT191_ALERT_3_G A Non-default SADI Restraint Value has been used     0.0400 Report
PLAT191_ALERT_3_G A Non-default SADI Restraint Value has been used     0.0400 Report
```

```
PLAT191_ALERT_3_G A Non-default SADI Restraint Value has been used     0.0400 Report
PLAT191_ALERT_3_G A Non-default SADI Restraint Value has been used     0.0400 Report
PLAT191_ALERT_3_G A Non-default SADI Restraint Value has been used     0.0400 Report
PLAT191_ALERT_3_G A Non-default SADI Restraint Value has been used     0.0400 Report
PLAT191_ALERT_3_G A Non-default SADI Restraint Value has been used     0.0400 Report
PLAT191_ALERT_3_G A Non-default SADI Restraint Value has been used     0.0400 Report
PLAT191_ALERT_3_G A Non-default SADI Restraint Value has been used     0.0400 Report
PLAT191_ALERT_3_G A Non-default SADI Restraint Value has been used     0.0400 Report
PLAT191_ALERT_3_G A Non-default SADI Restraint Value has been used     0.0400 Report
PLAT191_ALERT_3_G A Non-default SADI Restraint Value has been used     0.0400 Report
PLAT191_ALERT_3_G A Non-default SADI Restraint Value has been used     0.0400 Report
PLAT300_ALERT_4_G Atom Site Occupancy of C1A        Constrained at        0.5 Check
PLAT300_ALERT_4_G Atom Site Occupancy of C1B        Constrained at        0.5 Check
PLAT300_ALERT_4_G Atom Site Occupancy of C2A        Constrained at        0.5 Check
PLAT300_ALERT_4_G Atom Site Occupancy of C2B        Constrained at        0.5 Check
PLAT300_ALERT_4_G Atom Site Occupancy of C3A        Constrained at        0.5 Check
PLAT300_ALERT_4_G Atom Site Occupancy of C3B        Constrained at        0.5 Check
PLAT300_ALERT_4_G Atom Site Occupancy of C4A        Constrained at        0.5 Check
PLAT300_ALERT_4_G Atom Site Occupancy of C4B        Constrained at        0.5 Check
PLAT300_ALERT_4_G Atom Site Occupancy of C5A        Constrained at        0.5 Check
PLAT300_ALERT_4_G Atom Site Occupancy of C5B        Constrained at        0.5 Check
PLAT300_ALERT_4_G Atom Site Occupancy of C6A        Constrained at        0.5 Check
PLAT300_ALERT_4_G Atom Site Occupancy of C6B        Constrained at        0.5 Check
PLAT300_ALERT_4_G Atom Site Occupancy of C7A        Constrained at        0.5 Check
PLAT300_ALERT_4_G Atom Site Occupancy of C7B        Constrained at        0.5 Check
PLAT300_ALERT_4_G Atom Site Occupancy of C8A        Constrained at        0.5 Check
PLAT300_ALERT_4_G Atom Site Occupancy of C8B        Constrained at        0.5 Check
PLAT300_ALERT_4_G Atom Site Occupancy of H7BA       Constrained at        0.5 Check
PLAT300_ALERT_4_G Atom Site Occupancy of H1A        Constrained at        0.5 Check
PLAT300_ALERT_4_G Atom Site Occupancy of H1B        Constrained at        0.5 Check
PLAT300_ALERT_4_G Atom Site Occupancy of H7BB       Constrained at        0.5 Check
PLAT300_ALERT_4_G Atom Site Occupancy of H2A        Constrained at        0.5 Check
PLAT300_ALERT_4_G Atom Site Occupancy of H2B        Constrained at        0.5 Check
PLAT300_ALERT_4_G Atom Site Occupancy of H7BC       Constrained at        0.5 Check
PLAT300_ALERT_4_G Atom Site Occupancy of H8BA       Constrained at        0.5 Check
PLAT300_ALERT_4_G Atom Site Occupancy of H4A        Constrained at        0.5 Check
PLAT300_ALERT_4_G Atom Site Occupancy of H4B        Constrained at        0.5 Check
PLAT300_ALERT_4_G Atom Site Occupancy of H8BB       Constrained at        0.5 Check
PLAT300_ALERT_4_G Atom Site Occupancy of H8BC       Constrained at        0.5 Check
PLAT300_ALERT_4_G Atom Site Occupancy of H7AA       Constrained at        0.5 Check
PLAT300_ALERT_4_G Atom Site Occupancy of H7AB       Constrained at        0.5 Check
PLAT300_ALERT_4_G Atom Site Occupancy of H7AC       Constrained at        0.5 Check
PLAT300_ALERT_4_G Atom Site Occupancy of H8AA       Constrained at        0.5 Check
PLAT300_ALERT_4_G Atom Site Occupancy of H8AB       Constrained at        0.5 Check
PLAT300_ALERT_4_G Atom Site Occupancy of H8AC       Constrained at        0.5 Check
PLAT301_ALERT_3_G Main Residue  Disorder .............(Resd  1  )        34% Note
PLAT412_ALERT_2_G Short Intra XH3 .. XHn     H1B      ..H16A     .       2.05 Ang.
                                                        x,y,z  =      1_555 Check
PLAT412_ALERT_2_G Short Intra XH3 .. XHn     H8AA     ..H16C     .       1.96 Ang.
                                                    1-y,1-z,x  =     12_665 Check
PLAT606_ALERT_4_G Solvent Accessible VOID(S) in Structure .......          ! Info
PLAT720_ALERT_4_G Number of Unusual/Non-Standard Labels .........         12 Note
PLAT764_ALERT_4_G Overcomplete CIF Bond List Detected (Rep/Expd) .       1.37 Ratio
PLAT789_ALERT_4_G Atoms with Negative _atom_site_disorder_group  #         34 Check
PLAT811_ALERT_5_G No ADDSYM Analysis: Too Many Excluded Atoms ....          ! Info
PLAT860_ALERT_3_G Number of Least-Squares Restraints ............        498 Note
PLAT883_ALERT_1_G No Info/Value for _atom_sites_solution_primary .     Please Do !
```

```
PLAT909_ALERT_3_G Percentage of I>2sig(I) Data at Theta(Max) Still        74% Note
PLAT910_ALERT_3_G Missing # of FCF Reflection(s) Below Theta(Min).          1 Note
PLAT913_ALERT_3_G Missing # of Very Strong Reflections in FCF ....          1 Note
PLAT978_ALERT_2_G Number C-C Bonds with Positive Residual Density.          0 Info
```

```
   5 ALERT level A = Most likely a serious problem - resolve or explain
  23 ALERT level B = A potentially serious problem, consider carefully
  30 ALERT level C = Check. Ensure it is not caused by an omission or oversight
  78 ALERT level G = General information/check it is not something unexpected

   8 ALERT type 1 CIF construction/syntax error, inconsistent or missing data
  58 ALERT type 2 Indicator that the structure model may be wrong or deficient
  26 ALERT type 3 Indicator that the structure quality may be low
  43 ALERT type 4 Improvement, methodology, query or suggestion
   1 ALERT type 5 Informative message, check
```

It is advisable to attempt to resolve as many as possible of the alerts in all categories. Often the minor alerts point to easily fixed oversights, errors and omissions in your CIF or refinement strategy, so attention to these fine details can be worthwhile. In order to resolve some of the more serious problems it may be necessary to carry out additional measurements or structure refinements. However, the purpose of your study may justify the reported deviations and the more serious of these should normally be commented upon in the discussion or experimental section of a paper or in the "special_details" fields of the CIF. checkCIF was carefully designed to identify outliers and unusual parameters, but every test has its limitations and alerts that are not important in a particular case may appear. Conversely, the absence of alerts does not guarantee there are no aspects of the results needing attention. It is up to the individual to critically assess their own results and, if necessary, seek expert advice.

Publication of your CIF in IUCr journals

A basic structural check has been run on your CIF. These basic checks will be run on all CIFs submitted for publication in IUCr journals (*Acta Crystallographica*, *Journal of Applied Crystallography*, *Journal of Synchrotron Radiation*); however, if you intend to submit to *Acta Crystallographica Section C* or *E* or *IUCrData*, you should make sure that full publication checks are run on the final version of your CIF prior to submission.

Publication of your CIF in other journals

Please refer to the *Notes for Authors* of the relevant journal for any special instructions relating to CIF submission.

Validation response form

Please find below a validation response form (VRF) that can be filled in and pasted into your CIF.

```
# start Validation Reply Form
_vrf_CHEMW01_ca_b
;
PROBLEM: The difference between the given and expected weight for
```

```
RESPONSE: ...
;
_vrf_RINTA01_ca_b
;
PROBLEM: The value of Rint is greater than 0.12
RESPONSE: ...
;
_vrf_PLAT018_ca_b
;
PROBLEM: _diffrn_measured_fraction_theta_max .NE. *_full           ! Check
RESPONSE: ...
;
_vrf_PLAT906_ca_b
;
PROBLEM: Large K Value in the Analysis of Variance ......      7.116 Check
RESPONSE: ...
;
_vrf_PLAT971_ca_b
;
PROBLEM: Check Calcd Resid. Dens.  2.83Ang From C8A             2.14 eA-3
RESPONSE: ...
;
_vrf_PLAT977_ca_b
;
PROBLEM: Check Negative Difference Density on H15B      .      -0.39 eA-3
RESPONSE: ...
;
# end Validation Reply Form
```

PLATON version of 28/11/2022; check.def file version of 28/11/2022

Datablock ca_b - ellipsoid plot

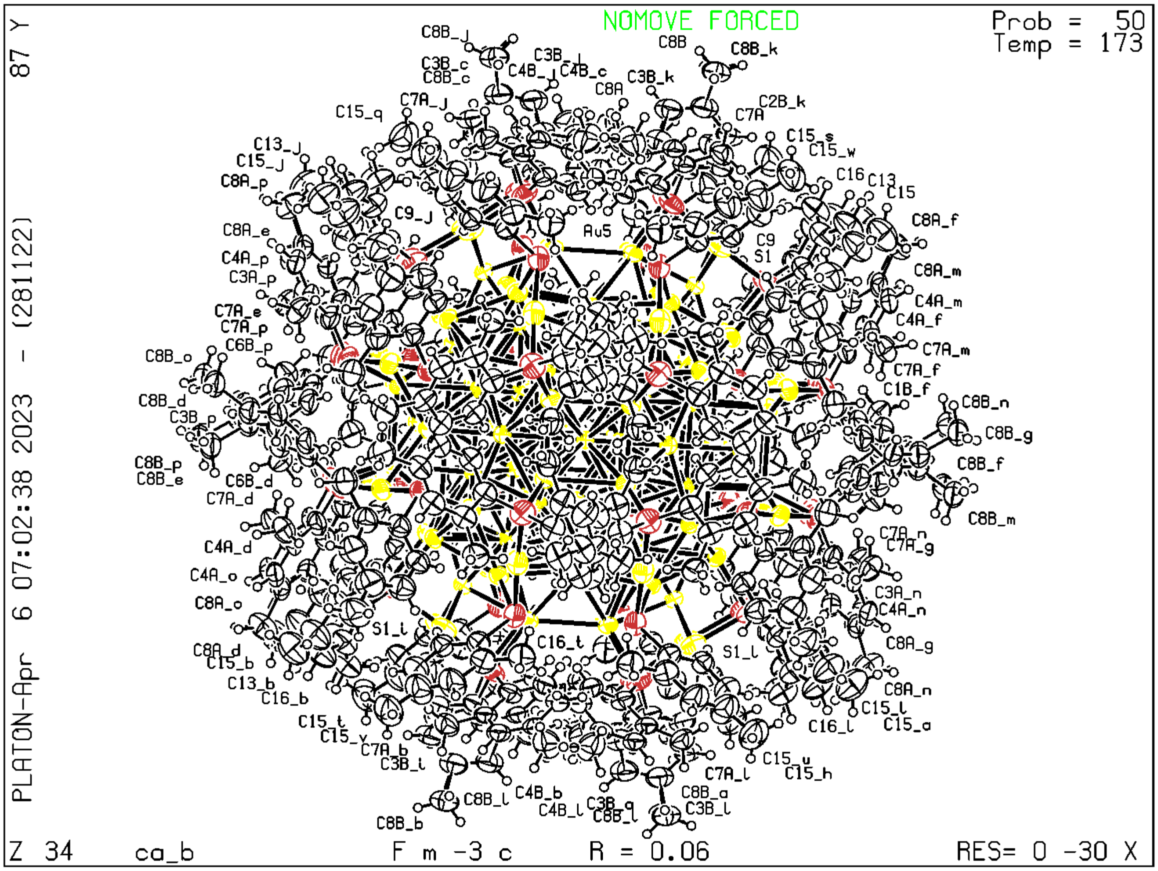

Supplement: Supplementary file 1 — Supplementary Information [file 41467_2023_38179_MOESM1_ESM.pdf]
